# Robust seismicity forecasting based on Bayesian parameter estimation for epidemiological spatio-temporal aftershock clustering models

**DOI:** 10.1038/s41598-017-09962-z

**Published:** 2017-08-29

**Authors:** Hossein Ebrahimian, Fatemeh Jalayer

**Affiliations:** 1 Post-doctoral Researcher, Department of Structures for Engineering and Architecture, University of Naples Federico II, Via Claudio 21, 80125 Naples, Italy; 2 Associate Professor, Department of Structures for Engineering and Architecture, University of Naples Federico II, Via Claudio 21, 80125 Naples, Italy

## Abstract

In the immediate aftermath of a strong earthquake and in the presence of an ongoing aftershock sequence, scientific advisories in terms of seismicity forecasts play quite a crucial role in emergency decision-making and risk mitigation. Epidemic Type Aftershock Sequence (ETAS) models are frequently used for forecasting the spatio-temporal evolution of seismicity in the short-term. We propose robust forecasting of seismicity based on ETAS model, by exploiting the link between Bayesian inference and Markov Chain Monte Carlo Simulation. The methodology considers the uncertainty not only in the model parameters, conditioned on the available catalogue of events occurred before the forecasting interval, but also the uncertainty in the sequence of events that are going to happen during the forecasting interval. We demonstrate the methodology by retrospective early forecasting of seismicity associated with the 2016 Amatrice seismic sequence activities in central Italy. We provide robust spatio-temporal short-term seismicity forecasts with various time intervals in the first few days elapsed after each of the three main events within the sequence, which can predict the seismicity within plus/minus two standard deviations from the mean estimate within the few hours elapsed after the main event.

## Introduction

Following a large earthquake and in the presence of a vast number of aftershocks, short-term operational seismicity forecasts (in the order of days to months) are of utmost importance for emergency decision-making and risk mitigation in the disaster area^1–6^. The aftershock activity is forecasted mainly based on the observed data of already registered (and mostly incomplete) recordings within the ongoing sequence^7^. The Epidemic Type Aftershock Sequence (ETAS) model^8, 9^ is the stochastic model most frequently used to describe earthquake occurrence within a seismic sequence^10^. It is an epidemic stochastic point process in which every earthquake within the sequence is a potential triggering event for subsequent earthquakes^11^, and therefore generates its own well-defined Modified Omori^12, 13^ (MO) aftershock decay^14^. Hence, it is capable of accounting for the triggering effect of all the events that have taken place before a desired time. The ETAS model performed quite well in operational seismic forecasting during the L’Aquila 2009 (central Italy) seismic sequence^15^. The model parameters are usually calibrated a priori based on the maximum likelihood criterion. The first effort on the calibration of temporal model parameters has been carried on by Ogata^8^, and extended later^9, 16–19^ to estimate the spatio-temporal model parameters. In addition, several attempts are made for developing improved algorithms to attain maximum likelihood estimates of ETAS parameters; e.g., the Expected-Maximization algorithm^20^, an improved maximum likelihood algorithm^21^, and a new algorithm based on Simulated Annealing optimization technique^10^, which allows for an automatic maximum likelihood estimation of the model parameters instead of fine-tuning of some algorithm parameters in advance. Adaptive model parameter estimation based on the events in the ongoing sequence (e.g., calibrating the parameters of MO and ETAS models based on the ongoing catalogue by employing Bayesian parameter estimation^22–25^) has the advantage of both tuning a sequence-specific model and also capturing possible time variations of the model parameters. As the original purpose of the present paper, we propose a fully simulation-based method to provide a *robust estimate* ^24, 26, 27^ for the spatial distribution of the events in a prescribed forecasting time interval after the main event. In the context of this robust estimate, the uncertainty in the ETAS model parameters is taken into account as the posterior joint probability distribution for the model parameters conditioned on the events that have already occurred (i.e., registered events in the ongoing seismic sequence before the beginning of the forecasting interval). The Markov Chain Monte Carlo (MCMC) simulation scheme^24, 25, 27^ is used to sample directly from the posterior probability distribution for ETAS model parameters (i.e., conditioned on the registered events in the ongoing sequence). Moreover, this robust estimate also considers the sequence of events that is going to occur during the forecasting interval (and hence affect the seismicity in an epidemic type model like ETAS). Although this sequence is unknown at the time of forecasting, we propose a stochastic procedure to generate it. The procedure leads to the stochastic spatio-temporal distribution of the forecasted events and consequently to the uncertainty in the estimated number of events, corresponding to a given forecasting interval. The resulting robust forecasts are directly applicable in adaptive daily aftershock hazard and risk assessment procedures^23, 28, 29, 30^.

**Figure 1:**
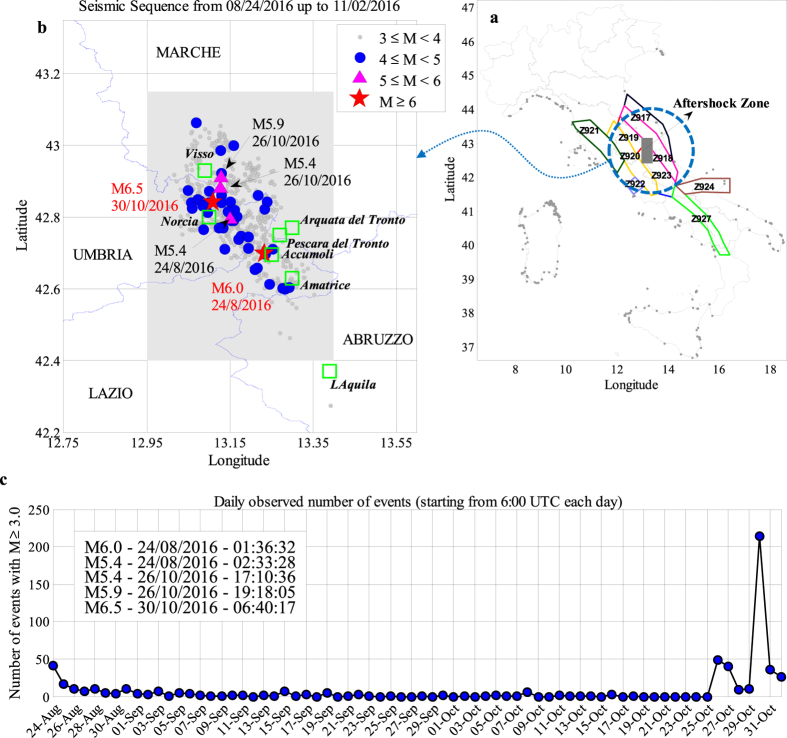
Amatrice 2016 seismic sequence. (**a**) The aftershock zone indicated by the grey-coloured box in perspective with the surrounding Italian seismogenic zonation. (**b**) The spatial distribution of aftershock events based on Catalogue 2 from August 24, 2016 (01:36 UTC) up to November 2, 2016 (10:32 UTC) bordering four neighbouring provinces in Italy. The grey-coloured box defines the considered aftershock zone and the most damaged towns are highlighted with green boxes. The main seismic events are illustrated as follows: M6.0 and M6.5 with red stars; M5.4 (24/08/16), M5.4 (26/10/16), M5.9 (26/10/16) with magenta triangles; aftershocks M ≥ 3.0 with grey circles. (**c**) The number of events (with M ≥ 3.0) in Catalogue 2 occurred within a 24-hour interval starting from 6:00 UTC of the desired day (MATLAB 2016b, http://softwaresso.unina.it/matlab/ is used to create this figure).

The proposed methodology is applied to provide retrospective forecasting for seismic activities of the 2016 Amatrice sequence by analysing the registered data of quasi real-time catalogues from INGV (Istituto Nazionale di Geofisica E Vulcanologia). The corresponding aftershock zone, as shown in Fig. 1a by the gray-colored area, is located mostly within the seismic zone 923 based on the ZS9 Italian Seismogenetic Zonation^31^. Fig. 1a shows also the seismogenic zonations surrounding the aftershock zone. It is to note that based on the Italian seismic zonations, the upper-bound magnitude for seismic zone Z923 is *M*_max_ = 7.06. On the 24th of August 2016 at 01:36 UTC, a Mw 6.0 earthquake struck the Central Italy between towns of Norcia and Amatrice, devastating Amatrice, Accumoli and several surrounding small towns and villages, causing almost 300 fatalities and leaving almost 30,000 homeless. The seismic sequence, including a Mw 5.4 aftershock (occurred almost one hour after the main shock at 02:33 UTC), triggered hundreds of earthquakes per day until the mid-September. Two months after, on the 26th of October, a Mw 5.4 followed within a two-hour delay by a Mw 5.9 earthquake (at 17:10 and 19:18 UTC, respectively) took place in the east of town Visso, and preceded the largest event of the sequence, a Mw 6.5 on October 30 at 06:40 UTC, North of Norcia^32^. This one is the largest earthquake recorded in Italy since the Mw 6.9 1980 Irpinia event. Fig. 1b and c illustrate the seismic activities within the aftershock zone during the first two months highlighting the key events taken place.

In the adaptive procedure presented in Section Method, the spatial maps can provide indications with regards to automatic re-sizing of the aftershock zone (if required) in case the spatial extension of the seismicity is not captured adequately inside a designated aftershock zone. For each forecasting interval, the spatial distribution of aftershock events is estimated based on equation (1) by calculating the (average) number of events *N*(*x*, *y*, *m*|**seq**, *M*_*l*_) in the cell unit centered at Cartesian coordinate (*x*, *y*) with magnitude greater than or equal to *m*. For a prescribed aftershock zone extension, the boundaries of the zone would need to be adjusted if *N*(*x*, *y*, *m*|**seq**, *M*_*l*_) is significantly greater than zero along the border cell units. This is a very important issue especially in case of Amatrice (multiple) seismic sequence 2016, where the seismicity was relocated towards north-west after two months from the main event on August 24. In this study, the aftershock zone is assigned a priori and the adequacy of its extension is monitored for each forecast.

The present study strives to perform robust forecasts for the spatio-temporal evolution of the events in specific time intervals within the very complex sequence described above that is distinguished by three main events (“mainshocks”) of moment magnitudes 6.0, 5.9, and 6.5, respectively (as illustrated in Fig. 1b). We divide the sequence into three parts: (a) from 24-August to 25-October, (b) from 26-October to 29-October, and finally (c) from 30-October to 1-November. We have used two different catalogues herein in order to gather data backwards in time. The two catalogues are distinguished by the date of access to the source database and share the time of origin (01:36 UTC, August 24 2016). The first one (labelled as Catalog 1) lasts until September 9, 2016 (07:23 UTC, date of first access) when the aftershock activities are reduced considerably in the zone. The second one (labelled as Catalog 2 whose registered data are illustrated in Fig. 1b and c) covers until November 2, 2016 (10:32 UTC, date of second access).

## Results

### Providing daily forecasts of seismicity from August 24 up to October 25

The prediction time window [*T*_*start*_, *T*_*end*_] indicates a 24-hour interval where *T*_*start*_ is 6:00 UTC of the following day. Each forecast uses available information at the time when the forecast is issued, i.e., the sequence (denoted as **seq** in Section Methods) comprised of events registered in the Catalogue 1 including the main event up to the time *T*_*start*_. To issue the first forecast, the observation history, **seq**, comprises the main event with Mw 6.0 at 01:36 UTC and the triggered events up to 6:00 UTC of 24 August 2016, where the lower cut-off magnitude, *M*_*l*_, of Catalogue 1 is equal to 3.0 based on the two methods discussed in Ebrahimian *et al*.^23^. It is to note that the completeness threshold of the catalog of aftershocks in subsequent forecasting intervals is demonstrated to be even lower than 3.0 (the procedure(s) adopted for evaluating the completeness magnitude *M*_*c*_ throughout the various phases of this multiple seismic sequence is described in detail in the Supplementary Information in a Section entitled “Discussion on the Completeness Magnitude *M*_*c*_”). In any case, *M*_*l*_ = 3.0 is considered as the cut-off threshold for the computation of the aftershock rates for the upcoming days.

The first step towards providing seismicity forecasts (with reference to equation 9) is sampling from the distribution of modal parameters **θ** based on posterior (target) probability distribution *p*(**θ**|**seq**, *M*_*l*_). The vector **θ** = [*β*, *K*, *K*_*t*_, *K*_*R*_, *c*, *p*, *d*, *q*] is updated on a daily basis by applying the Bayesian updating routine illustrated in equation (17) and considering that parameters *K*, *K*_*t*_, *K*_*R*_ are derived as function of other parameters within vector **θ** (see equations 4–7). As prior information, we assigned a normal distribution to the five model parameters [*β*, *c*, *p*, *d*, *q*], with a coefficient of variation (COV) equal to 0.30. The prior mean values for [*β*, *c*] are assigned equal to those provided by the MO Italian generic model parameters^33^, while prior mean values assigned to [*p*, *d*, *q*] are equal to the ETAS model parameters calibrated for the L’Aquila aftershock sequence^15^. The prior COV for each model parameter is set equal to 0.3 –in some cases larger than the reported prior COV’s in the above-mentioned references– to avoid using an over-informative prior distribution (i.e., a prior with a very low COV). Samples for **θ** are generated as a Markov Chain sequence directly from *p*(**θ**|**seq**, *M*_*l*_), as noted in Section Methods. It should be noted that sampled *p* and *q* values smaller than one are rejected according to equations (4) and (5) with the constraint *p* > 1 and *q* > 1. One key restraint on performing operational forecasting during an ongoing sequence is that the procedure should be performed in a reasonably small amount of time. To address this issue, an MCMC procedure for updating the model parameters is carried out adaptively (see the Section Methods).

The evolution in the statistics (mean and COV) of model parameters **θ** = [*β*, *K*, *K*_*t*_, *K*_*R*_, *c*, *p*, *d*, *q*] are summarized in Table 1. We use Catalogue 1 for constructing **seq** in order to provide forecasts within the first two weeks after the main event (i.e., from August 24 up to September 06). The first row of Table 1 corresponds to the statistics for the prior marginal PDF’s for **θ** while the subsequent rows indicate the statistics for the posterior distributions. In addition, Supplementary Fig. S1 illustrates the sampled histograms representing the marginal prior and posterior PDF’s corresponding to the six model parameters [*β*, *K*, *c*, *p*, *d*, *q*]. Moreover, we have illustrated the PDF for *K*, derived as a function of other model parameters, in Supplementary Fig. S1. The marginal PDF’s for the other two parameters *K*_*t*_ and *K*_*R*_ are not shown in the figure since they have a very straightforward relationship to other model parameters (see equations 4 and 5). The marginal distributions shown correspond to a 24 hours (1 day) forecasting interval and a magnitude 3.0 lower cut-off.

**Table 1:** Statistics (mean and COV) of ETAS model parameters **θ** for estimating the daily seismicity in the first 14 days elapsed after Mw 6.0 at 24-Aug. 2016 (Supplementary Fig. S1 also illustrates the sampled histograms representing the marginal posterior PDF’s corresponding to the six model parameters [*β*, *K*, *c*, *p*, *d*, *q*]).

	*β*		*c* [day]		*p*		*d* [km]		*q*		*K*		*K*_*t*_		*K*_*R*_	
	mean	COV	mean	COV	mean	COV	mean	COV	mean	COV	mean	COV	mean	COV	mean	COV
Prior	2.21	0.30	0.03	0.30	1.10	0.30	1.00	0.30	1.50	0.30						
24/08/2016	1.38	0.08	0.03	0.28	1.47	0.14	1.48	0.15	1.63	0.07	0.60	0.30	0.08	0.25	0.35	0.36
25/08/2016	1.53	0.07	0.03	0.27	1.44	0.08	1.60	0.11	1.70	0.06	0.35	0.17	0.08	0.14	0.47	0.43
26/08/2016	1.54	0.06	0.02	0.29	1.40	0.09	1.60	0.12	1.74	0.05	0.36	0.17	0.09	0.11	0.49	0.32
27/08/2016	1.57	0.06	0.02	0.28	1.40	0.08	1.49	0.11	1.72	0.05	0.33	0.15	0.09	0.11	0.43	0.31
28/08/2016	1.60	0.06	0.03	0.27	1.38	0.06	1.55	0.17	1.74	0.07	0.33	0.16	0.09	0.09	0.52	0.55
29/08/2016	1.59	0.06	0.03	0.30	1.34	0.10	1.54	0.12	1.75	0.05	0.36	0.19	0.09	0.08	0.48	0.35
30/08/2016	1.61	0.05	0.02	0.42	1.27	0.08	1.41	0.16	1.67	0.07	0.39	0.22	0.09	0.13	0.37	0.46
31/08/2016	1.64	0.05	0.02	0.30	1.24	0.06	1.53	0.14	1.74	0.06	0.38	0.21	0.09	0.11	0.48	0.39
01/09/2016	1.63	0.05	0.02	0.29	1.19	0.06	1.57	0.11	1.75	0.05	0.51	0.19	0.09	0.15	0.49	0.36
02/09/2016	1.64	0.04	0.02	0.33	1.18	0.06	1.60	0.15	1.76	0.06	0.49	0.24	0.08	0.21	0.54	0.42
03/09/2016	1.64	0.05	0.02	0.33	1.17	0.05	1.62	0.11	1.78	0.05	0.48	0.23	0.09	0.19	0.56	0.34
04/09/2016	1.63	0.05	0.02	0.24	1.19	0.05	1.52	0.11	1.73	0.04	0.46	0.20	0.09	0.14	0.45	0.31
05/09/2016	1.63	0.05	0.02	0.27	1.19	0.05	1.59	0.10	1.80	0.06	0.45	0.19	0.09	0.14	0.57	0.35
06/09/2016	1.65	0.05	0.02	0.29	1.17	0.05	1.57	0.09	1.75	0.04	0.46	0.23	0.09	0.16	0.49	0.27

**Figure 2:**
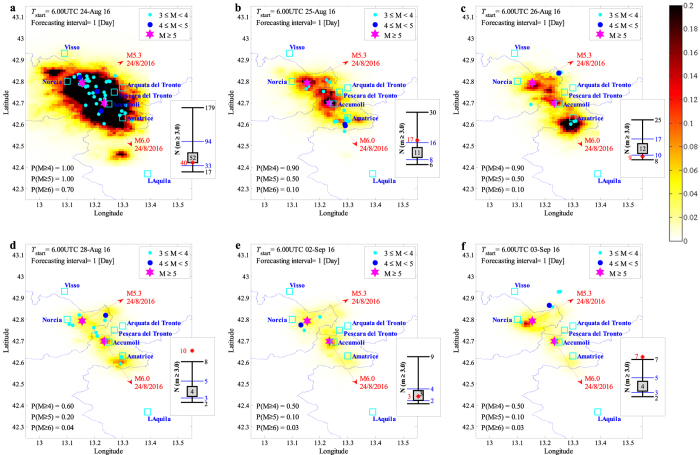
Forecasted vs. observed seismicity distribution in the aftershock zone, the maps report the mean +2 standard deviation confidence interval for the number of events per [km^2^] (latitude/longitude cells of a 0.01° × 0.01° grid) equal to or greater than magnitude *M*_*l*_ = 3 in the indicated 24-hour forecasting time window. In the lower left corner, the daily probabilities of having earthquakes with magnitudes 4, 5, and 6 or larger are reported. Each sub-plot also features the earthquakes (coloured dots) that occurred during the corresponding forecasting time window together with the two main events with *M* ≥ 5.0 (magenta stars). The sub-figures illustrate the observed (plotted in red star) vs. the error-bar for the forecasted number of events with *m* ≥ *M*_*l*_ corresponding to the forecasting time interval: the median value (the 50^th^ percentile, equivalent of the logarithmic mean in the arithmetic scale) inside a grey-filled square, the (logarithmic) mean plus/minus one (logarithmic) standard deviation indicating the interval between 16^th^ and 84^th^ percentiles (marked with blue horizontal lines), and the (logarithmic) mean plus/minus two (logarithmic) standard deviations indicating the interval between 2^nd^ and 98^th^ percentiles (marked with black horizontal lines). (MATLAB 2016b, http://softwaresso.unina.it/matlab/ is used to create this figure).

The sequence of events taking place during the forecasting interval, **seqg**, is generated (see equation 11 and Section Methods) given that **θ** and **seq** are known. Finally, substituting the sampled values for **θ** and **seqg** in equation (9), the robust estimate for the number of events with *M* ≥ *m* in a spatial cell units centered at (*x*, *y*) within the aftershock zone is obtained. This robust estimate is calculated as the expected number of events considering the uncertainties in the spatio-temporal distribution of the sequence of events. Fig. 2 shows the forecasted seismicity maps in terms of the mean plus two logarithmic standard deviation (98% confidence interval) for the number of events with *M* ≥ 3.0 within each spatial cell unit issued for the 24-hour time forecasting intervals. The earthquakes of interest occurred within the corresponding forecasting interval are illustrated as coloured dots (distinguished by magnitude). The two main events of the sequence with *M* ≥ 5.0 (see also Fig. 1) are identified with coloured stars (these events are shown for reference only and they did not necessarily take place in the illustrated map’s corresponding forecasting interval). We also report the forecasted daily probabilities of having earthquakes of magnitude equal to or larger than *m* = 4, 5 and 6 in the whole aftershock zone. These probabilities are calculated from the equation $1-{e}^{-N(m|{\bf{s}}{\bf{e}}{\bf{q}},{M}_{l})}$ where $N(m|{\bf{s}}{\bf{e}}{\bf{q}},{M}_{l})$ is the sum over the whole aftershock zone of the expected number of events $N(x,y,m|{\bf{s}}{\bf{e}}{\bf{q}},{M}_{l})$ from equation (9).

At the right-hand side of each sub-figure, the observed (shown as a red star) vs. forecasted number of events (shown in an error-bar format) is illustrated for events with *M*_*l*_ = 3.0 for the entire aftershock zone. The error-bar for the forecasted number of events features: the median value (the 50^th^ percentile, equivalent of the logarithmic mean in the arithmetic scale) inside a grey-filled square, the (logarithmic) mean plus/minus one (logarithmic) standard deviation indicating the interval between 16^th^ and 84^th^ percentiles (marked with blue horizontal lines), and the (logarithmic) mean plus/minus two (logarithmic) standard deviations indicating the interval between 2^nd^ and 98^th^ percentiles (marked with black horizontal lines). This is done to help in locating the observed number of events within plus or minus certain number of standard deviations from the mean estimate. It can be seen that the observed number of events lies within plus/minus one standard deviation of the mean estimate except for 28^th^ of August and 3^rd^ of September (for this one lies within two standard deviations away from the mean estimate).

### Providing daily forecasts of seismicity from October 26 up to October 29

As mentioned before, on 26th of October, a Mw 5.4, followed within a two-hour delay by a Mw 5.9 earthquake (at 17:10 and 19:18 UTC, respectively), took place in the east of town Visso (located in the north-western part of the aftershock zone, see Fig. 1b). This triggered a new aftershock sequence within the ongoing one. At this stage, given the time elapsed from the occurrence of the mainshock (i.e., around two months), it seemed quite tedious to consider all the events of interest up to the time of origin (i.e., 24^th^ of August) for each forecasting interval. To achieve this (i.e., avoid considering all the events back to 24^th^ of August), we performed a shift in the time of origin *T*_o_ from August 24^th^ to 17:10 UTC of October 26^th^ (time of occurrence of the Mw 5.4 earthquake, see Fig. 1). In the following, we describe the details of the forecasting procedure for the period from 26^th^ of October to 29^th^.

#### 24-hour forecasting from 6:00 UTC of 26/10/2016

**Figure 3:**
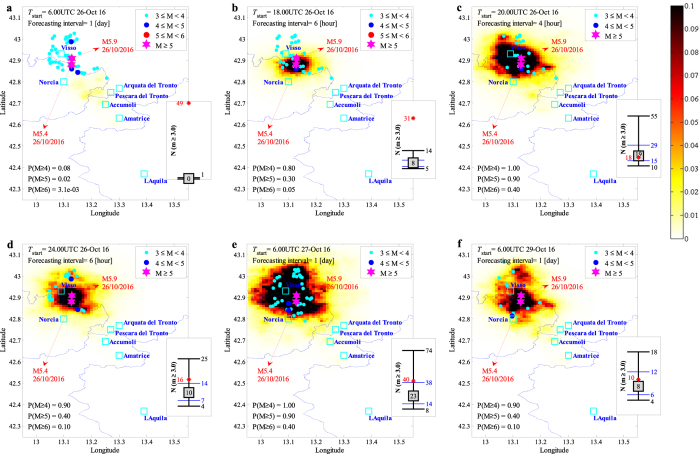
Forecasted vs. observed seismicity distribution in the aftershock zone, the maps report the mean + 2 standard deviation confidence interval for the number of events per [km^2^] (latitude/longitude cells of a 0.01° × 0.01° grid) equal to or greater than magnitude *M*_*l*_ = 3 in the indicated forecasting time window. In the lower left corner, the daily probabilities of having earthquakes with magnitudes 4, 5, and 6 or larger are reported. Each sub-plot also features the earthquakes (colored dots) that occurred during the corresponding forecasting time window together with the two main events with *M* ≥ 5.0 (magenta stars). The sub-figures illustrate the observed (plotted in red star) vs. the error-bar for the forecasted number of events with *m* ≥ *M*_*l*_ corresponding to the forecasting time interval: the median value (the 50^th^ percentile, equivalent of the logarithmic mean in the arithmetic scale) inside a grey-filled square, the (logarithmic) mean plus/minus one (logarithmic) standard deviation indicating the interval between 16^th^ and 84^th^ percentiles (marked with blue horizontal lines), and the (logarithmic) mean plus/minus two (logarithmic) standard deviations indicating the interval between 2^nd^ and 98^th^ percentiles (marked with black horizontal lines). (MATLAB 2016b, http://softwaresso.unina.it/matlab/ is used to create this figure).

We performed the forecasting in this period considering as the sequence **seq** all the events with magnitude greater than or equal to 3.0 that took place after 01:36 UTC of 24/08/2016 up to *T*_*start*_ which is 6:00 UTC of 26/10/2016 (including the main event of August 24^th^). To facilitate the ETAS model parameter **θ** estimation, we employed as prior distribution (instead of the priors considered in the previous section) the updated distribution of model parameters on 06-September (see the last row of Table 1); assuming that the model parameters did not undergo significant changes from 6^th^ of September to 26^th^ of October (the sequence activity has decayed significantly in this period). The MCMC procedure is carried out as described in the previous section. The statistics of the updated model parameters (mean and COV) are reported in the first row of Table 2. It can be observed (comparing with the prior distribution in the last row of Table 1) that model parameters remain more-or-less invariant (we could have directly used the ETAS model parameters estimated for 6^th^ of September). Fig. 3a illustrates the forecasted seismicity (mean plus two standard deviations) and the events of interest that occurred during the 24-hour interval of interest. The estimated number of events within the aftershock zone (shown in the error-bar plot reported in the right-hand side of the figure) shows a substantial reduction in the seismicity compared to the first days elapsed after the main event in August 24. The exceedance probabilities are calculated as *P*(*M* ≥ 4) = 0.08, *P*(*M* ≥ 5) = 0.02, and *P*(*M* ≥ 6) = 0.003. It is interesting to note that these probabilities are substantially higher than those provided from the calculation of the long-term seismicity level based on the ZS9 Italian Seismogenic Zonation^31^ data, estimating a daily probability of around 3.83 × 10^−4^ for *M* ≥ 4.76. Hence, the level of (forecasted) seismicity at the desired date is more than 40 times higher than the base seismicity level for *M* ≥ 5. The distribution of seismicity forecasted for 26^th^ of October (shown in Fig. 3a) is used next as an estimate of the background (base) seismicity denoted as *N*_*b*_(*x*, *y*, *m*|*M*_*l*_) in Section Methods.

**Table 2:** Statistics (mean and COV) of ETAS model parameters **θ** for estimating the daily seismicity rate of the desired dates for the second part of the sequence after Mw 5.4 at 26-October 2016 (Supplementary Fig. S2 also illustrates the sampled histograms representing the marginal posterior PDF’s corresponding to the six model parameters [*β*, *K*, *c*, *p*, *d*, *q*]).

	*β*		*c* [day]		*p*		*d* [km]		*q*		*K*		*K*_*t*_		*K*_*R*_	
	mean	COV	mean	COV	mean	COV	mean	COV	mean	COV	mean	COV	mean	COV	mean	COV
26/10/2016^*^	1.67	0.05	0.02	0.36	1.17	0.06	1.57	0.12	1.74	0.06	0.56	0.26	0.08	0.23	0.47	0.31
26/10/2016^†^	1.34	0.17	0.03	0.27	1.20	0.14	1.39	0.17	1.55	0.09	9.48	0.20	0.08	0.46	0.28	0.43
26/10/2016‡	1.53	0.13	0.03	0.27	1.38	0.16	1.53	0.15	1.58	0.08	0.70	0.39	0.08	0.29	0.32	0.46
27/10/2016	1.35	0.10	0.03	0.26	1.38	0.14	1.51	0.13	1.69	0.08	1.33	0.19	0.09	0.23	0.43	0.41
29/10/2016	1.49	0.07	0.03	0.27	1.27	0.09	1.58	0.13	1.71	0.06	0.57	0.34	0.10	0.14	0.47	0.37

^*^Corresponding to Fig. 3(a,b); ^†^corresponding to Fig. 3c; ^‡^corresponding to Fig. 3d.

#### 6-hour forecasting from 18:00 UTC of 26/10/2016

**Figure 4:**
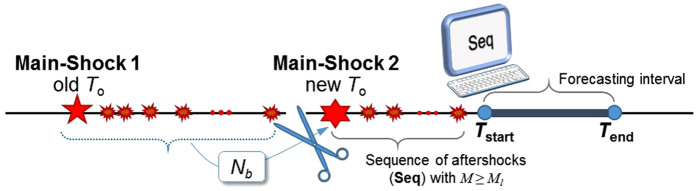
Schematic sketch of the shift in the time of origin *T*_o_.

After the occurrence of the event with Mw 5.4 at 17:10 UTC of 26/10/2016, we provide a 6-hour prediction of seismicity for the forecasting interval starting from *T*_*start*_ set to 18:00 UTC of 26/10/2016 (i.e., 50 minutes after the occurrence of Mw 5.4 event). At this point, we performed a shift in the time of origin by setting *T*_o_ to 17:10 UTC of 26^th^ of October (see Fig. 4). The sequence **seq** includes all the triggered events with *M* ≥ 3 occurred after 17:10 UTC of 26/10/2016 (including the main Mw 5.4 event). It should be noted that the event Mw 5.4 was not preceded by any foreshocks (i.e., no *M* ≥ 3 events took place between 06:00 UTC and 17:10 UTC of 26 of October). Given the very low seismic activity prior to the major event and given the presence of very few events in **seq**, we did not perform Bayesian updating on the model parameter **θ** and used the statistics provided in the first row of Table 2 issued for 26/10/2016 (the parameters updated in the previous step). It is important to note that the forecasted seismicity for the 24-hour interval elapsed after 06:00 UTC of October 26 in the previous step (shown in Fig. 3a) is used herein (after proportioning it to a 6-hour forecasting interval) as the background seismicity *N*_*b*_(*x*, *y*, *m*|*M*_*l*_). The background seismicity usually considers the long-term seismicity in the calculations and was assumed to be equal to zero in our previous calculations for the first part of the sequence staring from 24^th^ of August. Herein, we use this background seismicity to conservatively approximate the triggering effect of the events occurred in the first part of the sequence (from August 24^th^ to October 26^th^). The background seismicity is added as a constant term to the contribution of the triggering events (see Section Method). The forecasted seismicity map in terms of the mean plus two standard deviation for the number of events with *M* ≥ 3.0 is shown in Fig. 3b. Observed events with *M* ≥ 3.0 (coloured dots) occurred within the corresponding 6-hour forecasting interval are also highlighted on the map. The main two events with Mw 5.4 at 17:10 UTC assigned as the mainshock and the Mw 5.9 event at 19:18 UTC (which lies within the 6-hour forecasting interval) are shown with magenta stars. According to the right-hand side error-bar plot of Fig. 3b, the total number of registered events within the 6-hour forecasting interval (red star) is significantly higher than the forecasted values. This can be attributed to very few number of observed input data in **seq** for preforming the robust estimation and to the fact that model parameters were not tuned to the newly triggered sequence. Although less successful in predicting the number of events, the model predicts exceedance probabilities *P*(*M* ≥ 5) and *P*(*M* ≥ 6) to be more than 15 times compared to the initial estimates in Fig. 3a.

#### 4-hour forecasting from 20:00 UTC of 26/10/2016

After the occurrence of the event with Mw 5.9 at 19:18 UTC, the seismicity forecast is provided again for the interval starting from 20:00 UTC (42 minutes after the Mw 5.9 event) up to 24:00 UTC of 26/10/2016 (i.e., a 4-hour time interval). The corresponding **seq** includes all the events with *M* ≥ 3.0 which occurred (including the main event of Mw 5.4 at 17:10 UTC and Mw 5.9 at 19:18 UTC) after 17:10 UTC up to the starting time (20:00 UTC) of October 26. The model parameters **θ** are updated based on the information provided by the sequence **seq** with *M*_*l*_ set to 2.5 and reported in the second row of Table 2. Note that the cut-off magnitude lower than 3.0 is assigned only for model updating purposes to gain more data and the seismicity rate is later calculated with *M*_*l*_ = 3.0. Fig. 3c illustrates the forecasted seismicity map in terms of the mean plus two standard deviation for the number of events with *M* ≥ 3.0 within the 4-hour forecasting interval. Note that for the 4-hour time interval, the exceedance probabilities *P*(*M* ≥ 5) and *P*(*M* ≥ 6) increase considerably in Fig. 3c after the occurrence of the event with Mw 5.9 at 19:18 UTC (compared to Fig. 3a). In addition, the observed number of events within the 4-hour time interval (sub-figure) lies within the plus/minus one standard deviation confidence interval.

#### 6-hour forecasting from 24:00 UTC of October 26, and 24-hour forecasting from 6:00 UTC of 27^th^ and 29^th^ of October

At this stage, seismicity forecasts are provided first for the 6-hour time interval starting from 24:00 UTC of 26/10/2016, and later extended for issuing 24-hour forecasts with *T*_*start*_ = 6:00UTC corresponding to the two consecutive days of October 27 and October 29. These predictions are issued based on the sequence of events **seq** including the mainshock of Mw 5.4 (considering 17:10 UTC 26^th^ of October as time of origin *T*_*o*_) and the triggered events with *M* ≥ 3.0 up to the associated *T*_*start*_. The last three rows of Table 2 illustrate the statistics of updated model parameters. The predicted seismicity distribution maps and the corresponding error-bar plots for the number of events (in sub-plots) are presented in Fig. 3(d–f), which manage to properly forecast the distribution/number of observed events.

#### Providing daily forecasts of seismicity from October 30 up to November 1

As mentioned before, on 30th of October, a Mw 6.5 event occurred in the North of Norcia at 6:40 UTC (located in the north-western part of the aftershock zone, see Fig. 1b).

#### 24-hour forecasting from 6:00 UTC of 30^th^ of October (starting time 40 minutes before the main event)

**Figure 5:**
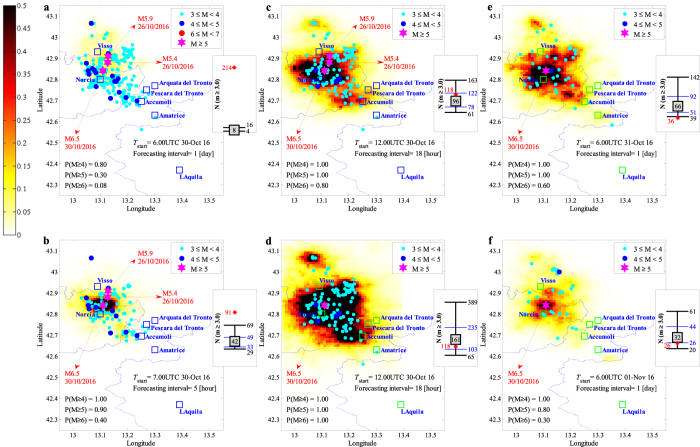
Forecasted vs. observed seismicity distribution in the aftershock zone, the maps report the mean + 2 standard deviation confidence interval for the number of events per [km^2^] (latitude/longitude cells of a 0.01° × 0.01° grid) equal to or greater than magnitude *M*_*l*_ = 3 in the indicated forecasting time window. In the lower left corner, the daily probabilities of having earthquakes with magnitudes 4, 5, and 6 or larger are reported. Each sub-plot also features the earthquakes (colored dots) that occurred during the corresponding forecasting time window together with the main events with *M* ≥ 5.0 (magenta stars). The sub-figures illustrate the observed (plotted in red star) vs. the error-bar for the forecasted number of events with *m* ≥ *M*_*l*_ corresponding to the forecasting time interval: the median value (the 50^th^ percentile, equivalent of the logarithmic mean in the arithmetic scale) inside a grey-filled square, the (logarithmic) mean plus/minus one (logarithmic) standard deviation indicating the interval between 16^th^ and 84^th^ percentiles (marked with blue horizontal lines), and the (logarithmic) mean plus/minus two (logarithmic) standard deviations indicating the interval between 2^nd^ and 98^th^ percentiles (marked with black horizontal lines). (MATLAB 2016b, http://softwaresso.unina.it/matlab/ is used to create this figure).

The forecasted seismicity is issued for a 24-hour time interval starting at 6:00UTC of 30/10/2016 based on the sequence of events **seq** comprising of events occurring after the time of origin *T*_o_ set to 17:10 UTC of 26^th^ of October (time of occurrence of the Mw5.4 event). The statistics of the updated model parameters are shown in the first row of Table 3. The forecasted seismicity distribution for the next 24 hours for *M* ≥ 3 is mapped in Fig. 5a. The background seismicity is set to the time-invariant distributed seismicity equal to *N*_*b*_(*x*, *y*, *m*|*M*_*l*_) as defined in the previous section (shown in Fig. 3a). According to Fig. 5a, our blind prediction of the exceedance probability *P*(*M* ≥ 6) is a value around 10% which is quite high as compared to the daily probability equal to 3.83 × 10^−4^ for *M* ≥ 4.76 calculated based on long-term seismicity in the previous section. It is also interesting to note that the forecasted value for *P*(*M* ≥ 6) is equal to the forecast provided for October 29; this can be viewed as somewhat alarming (does not represent the expected decay with time). Nevertheless, the forecasted error-bar for the number of events (see right-hand side of Fig. 5a) is not able to properly predict the huge number of events triggered due to Mw 6.5 at 06:40UTC.

**Table 3:** Statistics (mean and COV) of ETAS model parameters **θ** for estimating the daily seismicity rate of the desired dates for the third part of the sequence after Mw 6.5 at 30-October 2016 (Supplementary Fig. S3 also illustrates the sampled histograms representing the marginal posterior PDF’s corresponding to the six model parameters [*β*, *K*, *c*, *p*, *d*, *q*]).

	*β*		*c* [day]		*p*		*d* [km]		*q*		*K*		*K*_*t*_		*K*_*R*_	
	mean	COV	mean	COV	mean	COV	mean	COV	mean	COV	mean	COV	mean	COV	mean	COV
30/10/2016^*^	1.52	0.06	0.03	0.27	1.22	0.08	1.67	0.13	1.76	0.06	0.70	0.22	0.09	0.16	0.58	0.40
30/10/2016^†^	1.42	0.06	0.03	0.26	1.17	0.07	1.64	0.12	1.70	0.06	1.16	0.26	0.08	0.30	0.48	0.36
30/10/2016^‡^	1.39	0.04	0.03	0.26	1.25	0.08	1.94	0.11	1.81	0.06	0.71	0.23	0.10	0.11	0.81	0.36
30/10/2016^††^	1.29	0.06	0.03	0.23	1.24	0.14	1.61	0.13	1.63	0.06	3.09	0.39	0.08	0.38	0.38	0.35
31/10/2016	1.44	0.03	0.04	0.21	1.31	0.10	1.61	0.11	1.71	0.04	0.69	0.25	0.10	0.11	0.47	0.29
01/11/2016	1.50	0.03	0.04	0.20	1.33	0.08	1.73	0.09	1.73	0.04	0.54	0.20	0.11	0.08	0.54	0.27

^*^Corresponding to Fig. 5a; ^†^corresponding to Fig. 5b; ^‡^corresponding to Fig. 5c; ^††^corresponding to Fig. 5d.

#### 5-hour forecasting from 7:00 UTC of 30^th^ of October (starting time 20 minutes after the main event of Mw 6.5)

In order to test the forecasting ability of the model right after the main event, we provided a forecast with starting time *T*_*start*_ set to 7:00UTC (only 20 minutes after the main event). The same as the previous forecast, the time of origin is set to 17.10 UTC of 26^th^ of October and the same invariant background seismicity *N*_*b*_(*x*, *y*, *m*|*M*_*l*_) is being adopted. The second row of Table 3 shows the statistics of the updated model parameters. The forecasted (5-hour) seismicity distribution is mapped in Fig. 5b. Moreover, the error-bar plot for the forecasted number of events is reported against the observed number of events. Comparing Fig. 5b with Fig. 5a, we can appreciate the improvement in the forecasted seismicity distribution and number of events based on the few events that occurred in the one hour that separates the two starting times (i.e., 6:00 UTC and 7:00 UTC, respectively).

#### 18-hour forecasting from 12:00 UTC of 30^th^ of October (starting time 5 hours and 20 minutes after the main event of Mw 6.5, *T*_o_ = 6:40 UTC of 30^th^ of October)

The next forecasting is performed for the same day of 30/10/2016 with *T*_*start*_ set to 12:00UTC and *T*_*end*_ set to 06:00 UTC of 31/10/2016 (i.e., 18-hour interval). At this stage, we performed a shift in the time of origin *T*_o_ from 17:10 UTC of 26^th^ of October to 6:40 UTC of 30^th^ of October. The background seismicity *N*_*b*_(*x*, *y*, *m*|*M*_*l*_) is set (and proportioned to an 18-hour interval) to that of 30^th^ of October for a 24-hour interval with starting time set to 6:00 UTC (shown in Fig. 5a). The prior distributions for the model parameters are taken equal to those of the original priors reported in the first row of Table 1. The fourth row of Table 3 shows the statistics of the updated model parameters. Fig. 5d shows the map of forecasted seismicity with the back-drop of events occurred in this interval. The error-bar plot for the forecasted number of events manages to capture the observed number of events within one standard deviation confidence interval.

#### 18-hour forecasting from 12:00 UTC of 30^th^ of October (starting time 5 hours and 20 minutes after the main event of Mw 6.5, *T*_o_ = 17:10 UTC of 26^th^ of October)

To measure the effect of the shift in the time of origin, the same forecasting presented in the previous step is performed with time of origin set to 17:40 UTC of 26^th^ of October. The third row of Table 3 illustrates the statistics of updated model parameters. Fig. 5c shows the forecasted map of seismicity and the error-bar for the predicted number of events. The forecasted number of events are slightly lower than those predicted in the previous step in Fig. 5c (after shifting the time of origin). This is to be expected since the latter forecast employs a time-invariant background seismicity to consider the events of interest occurred in the time interval between 17:10 UTC of 26^th^ of October and 6.40 UTC of 30^th^ of October. This is while the former forecast explicitly considers the triggering contribution of these events and the associated time-decay. Overall, it is reassuring to note that the two forecasts provide essentially the same information.

#### 24-hour forecasting from 6:00 UTC of October 31^st^ and November 1^st^

The seismicity forecasts are provided next for the 24-hour time intervals starting from 6:00UTC of October 31 and November 1. These predictions are issued based on time of origin *T*_o_ set to 6:40 UTC of 30 October and the sequence of records **seq** including all the events of interest *M* ≥ 3.0 that occurred between the time of origin *T*_o_ (including the 6.5 Mw “mainshock”) and the time of start *T*_*start*_ of each forecasting interval (6:00 UTC of each of these two days). The prior probability distributions are set to the original priors (whose statistics are reported in the first row of Table 1). The last two rows of Table 3 illustrate the statistics of updated model parameters. The seismicity forecasts (i.e, mean estimate plus two standard deviations) are shown in Fig. 5(e,f). The observed number of events lies within two standard deviation from the forecasted mean estimate.

## Discussion

We have proposed a fully simulation-based procedure for both Bayesian model updating of an epidemiological-type aftershock spatio-temporal clustering model and robust operational forecasting of the number of events of interest expected to happen in each time frame. The adopted epidemiological model parameters are defined so that the model converges asymptotically (spatio-temporally speaking) to the long-term seismicity of the zone of interest. The forecasting is “robust” because it considers the uncertainty (i.e., the joint probability distribution) in the model parameters. Apart from being quite efficient (the most challenging forecasting we performed took 45 minutes on a normal PC), the model updating and forecasting procedure is carried on without human interference and use of expert judgement. The model is simply “tuning-in” automatically into the sequence of observed events. The choice of the recent Central Italy sequence of events as a demonstration of this procedure, albeit quite a natural choice, proved to be very challenging. This is because the sequence embedded three “sub-sequences” with different productive and decaying properties. We used the peculiarities of this sequence to test several different strategies for forecasting. For example, we performed a shift in the time of origin of the sequence by conservatively introducing a constant background seismicity (calculated by the procedure). This shift proved to be quite useful as it relieved us from the burden of summing up the triggering properties of all the events that took place in the previous “sub-sequence” (or the previous part of the sequence as we may wish to call it) at the small price of neglecting the time-decay in their triggering contribution. We observe that after an initial transition time (in the order of few hours, enough to accumulate sufficient events for updating the model parameters), the model quickly tunes into the sequence and provides forecasting that is reliable in most cases up to plus/minus one standard deviations. As expected, the procedure falls short of predicting the “mainshock” of 26^th^ of October (17:10 UTC) as it happened when the sequence had decayed. The procedure, however, did a better job for forecasting the events occurred at 19:18 UTC of 26^th^ of October and on 6.40 UTC of 30^th^ October. This relative success can be attributed to the fact that these events took place at the initial stages of the newly triggered sequence of 26^th^ of October when the seismic activity was still very high. The estimated model parameters present some time-dependent fluctuations but after a certain number of days elapsed after the main event, they seem to stabilize. In general, the first sub-sequence (Mw 6 “mainshock” occurred at 1:36 UTC 24^th^ of August) seems to be the mildest one in terms of the time decay in seismicity and is the least active in terms of sequence’s productivity. The second sub-sequence (Mw 5.4 “mainshock” occurred at 17:10 UTC 26^th^ of October) is intermediate both in terms of the rate of time-decay and the productivity. The third sub-sequence (Mw 6.5 “mainshock” occurred at 6:40 UTC 30^th^ of October) has the steepest time-decay of seismicity and is the most active in terms of the productivity of the sequence. Last but not least, it is important to mention that the proposed procedure for robust forecasting is conditioned on the available catalogue of events and the epidemiological model adopted for capturing the spatio-temporal aftershock clustering.

## Methods

Let the aftershock occurrence be described by a non-homogenous Poisson point process over the two-dimensional space and time. Hence, the aftershock zone can be described as the set ***A*** in the Cartesian space discretized into mutually exclusive and collectively exhaustive (MECE) subsets or spatial cell units centered at (*x*, *y*) ∈ ***A***. In this manner, *λ*(*t*, *x*, *y*, *m*|**seq**, *M*_*l*_) represents the rate of occurrence of events in the forecasting interval [*T*_*start*_, *T*_*end*_] at time *t* elapsed after the main event (a.k.a. mainshock) occurred at time of origin *T*_o_ with magnitude greater than or equal to *m* and in the cell unit centered at (*x*, *y*) ∈ ***A***, given (a) the observation history **seq** which is the sequence of *N*_o_ events (including mainshock and the sequence of aftershocks) taken place before the forecasting interval (i.e. in the interval [*T*_o_, *T*_*start*_)), and (b) the lower cut-off magnitude *M*_*l*_. Hence, **seq** can be expressed as **seq** = {(*t*_*i*_, *x*_*i*_, *y*_*i*_, *m*_*i*_), *t*_*i*_ < *T*_*start*_, *m*_*i*_ ≥ *M*_*l*_, *i* = 1: *N*_o_}, where *t*_*i*_ is the arrival time for the *i*th event with magnitude *M*_*i*_ and location (*x*_*i*_, *y*_*i*_) ∈ ***A***. The average number of events in the spatial cell unit centered at (*x*, *y*) with magnitude greater than or equal to *m* in the forecasting interval [*T*_*start*_, *T*_*end*_] can then be calculated as:

1 $$N(x,y,m|{\bf{s}}{\bf{e}}{\bf{q}},{M}_{l})={N}_{b}(x,y,m|{M}_{l})+{\int }_{{T}_{start}}^{{T}_{end}}\lambda (t,x,y,m|{\bf{s}}{\bf{e}}{\bf{q}},{M}_{l}){\rm{d}}t$$

where *N*_*b*_(*x*, *y*, *m*|*M*_*l*_) is a constant representing the background seismicity of the area. Let θ denote the vector of model parameters for *λ*(*t*, *x*, *y*, *m*|**seq**, *M*_*l*_). Given a particular space-time model and a realization of the vector of model parameters θ, one can calculate a plausible value for the rate of occurrence denoted as *λ*(*t*, *x*, *y*, *m*|**θ**, **seq**, *M*_*l*_). Note that we have not included the conditioning on the model assumptions^34^ for the sake of brevity. A robust estimate^24, 26, 27, 35, 36^ of the average number of events in the spatial cell unit centered at (*x*, *y*) with magnitude greater than or equal to *m* in the forecasting interval [*T*_*start*_, *T*_*end*_], and over the domain of the model parameters **Ω**_**θ**_ can be calculated as:

2 $${\mathbb{E}}[N(x,y,m|{\bf{s}}{\bf{e}}{\bf{q}},{M}_{l})]={N}_{b}(x,y,m|{M}_{l})+{\int }_{{\Omega }_{{\boldsymbol{\theta }}}}{\int }_{{T}_{start}}^{{T}_{end}}\lambda (t,x,y,m|{\boldsymbol{\theta }},{\bf{s}}{\bf{e}}{\bf{q}},{M}_{l})\cdot p({\boldsymbol{\theta }}|{\bf{s}}{\bf{e}}{\bf{q}},{M}_{l}){\rm{d}}t{\rm{d}}{\boldsymbol{\theta }}$$

where *p*(**θ**|**seq**, *M*_*l*_) is the conditional probability distribution function (PDF) for **θ** given the **seq** and the lower cut-off magnitude *M*_*l*_.

### An epidemiological model for space-time clustering of aftershocks

The ETAS model is an epidemiological stochastic point process in which every earthquake is a potential triggering event for subsequent earthquakes^8, 9, 16–19^. According to the general ETAS model, we adopt the spatio-temporal triggering effect of a given sequence on the seismicity rate, denoted as *λ*_ETAS_, as:

3 $${\lambda }_{{\rm{ETAS}}}(t,x,y,m|{\boldsymbol{\theta }},{\bf{s}}{\bf{e}}{{\bf{q}}}_{t},{M}_{l})={e}^{-\beta (m-{M}_{l})}\sum _{{t}_{j} < t}K\,{e}^{\beta ({M}_{j}-{M}_{l})}\cdot \frac{{K}_{t}}{{(t-{t}_{j}+c)}^{p}}\cdot \frac{{K}_{R}}{{({r}_{j}^{2}+{d}^{2})}^{q}}$$

where **seq**_*t*_ = {(*t*_*j*_, *x*_*j*_, *y*_*j*_, *M*_*j*_), *t*_*j*_ < *t*, *M*_*j*_ ≥ *M*_*l*_} is the observation history up to the time *t*; parameter *β* is related to Gutenberg-Richter seismicity; parameters *c* and *p* are similar to those of the Modified Omori’s Law^12, 13^ defining the decay in time of short-term triggering effect; *d* and *q* characterize the spatial distribution of the triggered events; *r*_*j*_ is the distance between the location (*x*, *y*) ∈ *A* and the epicenter of the *j*th event (*x*_*j*_, *y*_*j*_); parameters *K*, *K*_*t*_ and *K*_*R*_ satisfy the achievement of asymptotic compatibility between ETAS predictions and the long-term seismicity. Thus, the vector of model parameters *λ* can be defined as **θ** = [*β*, *K*, *K*_*t*_, *K*_*R*_, *c*, *p*, *d*, *q*]. The integral of λ_ETAS_ over infinite space and time needs to converge in limit to the number of events predicted by the Gutenberg and Richter model with magnitude greater than *m*, denoted generally as *K*_o_ *e* ^−^^β^ ^*m*^; note that *K*_o_ is different from *K*. Herein, such compatibility is achieved by making sure that the following three conditions are satisfied:

1. The normalizing coefficient *K*_*t*_ is obtained such that integrating the time-dependent term over infinite time will in limit be equal to unity (see also Lippiello *et al*.^21, 37^):
  4 $${K}_{t}{\int }_{{t}_{j}}^{+\infty }\frac{{\rm{d}}t}{{(t-{t}_{j}+c)}^{p}}=1\therefore p > 1,{K}_{t}=(p-1){c}^{p-1}$$
2. The coefficient KR is normalized such that integrating the spatial term over infinite space will in limit be equal to unity (see also Lippiello *et al*.^21, 37^):
  5 $${\int }_{{x}_{j}}^{+\infty }{\int }_{{y}_{j}}^{+\infty }\frac{{K}_{R}{\rm{d}}x{\rm{d}}y}{{({(x-{x}_{j})}^{2}+{(y-{y}_{j})}^{2}+{d}^{2})}^{q}}\triangleq {K}_{R}{\int }_{0}^{+\infty }\frac{2\pi r{\rm{d}}r}{{[{r}^{2}+{d}^{2}]}^{q}}=1\therefore q > 1,{K}_{R}=\frac{(q-1)}{\pi }{d}^{2(q-1)}$$
3. The coefficient *K* is calibrated such that the number of events with magnitude greater than or equal to *M*_*l*_ taking place in time interval *t* ∈ [*T*_o_, *T*_*start*_] over the whole aftershock zone *A* is equal to *N*_*o*_:

6 $${\int }_{{T}_{{\rm{o}}}}^{{T}_{start}}{\iint }_{(x,y)\in {\boldsymbol{A}}}{\lambda }_{{\rm{ETAS}}}(t,x,y,{M}_{l}|{\boldsymbol{\theta }},{\bf{s}}{\bf{e}}{{\bf{q}}}_{t},{M}_{l}){\rm{d}}x{\rm{d}}y\,{\rm{d}}t={N}_{{\rm{o}}}$$

where *T*_o_ defines the mainshock occurrence time; the term *λ*_ETAS_(*t*, *x*, *y*, *M*_*l*_|θ, **seq**_*t*_, *M*_*l*_) is obtained by substituting *m* = *M*_*l*_ in equation (3), and denotes the rate of events with magnitude greater than or equal to *M*_*l*_. Since *N*_o_ denotes the events occurred within the aftershock zone ***A***, the integration over the whole aftershock zone can be approximated with that over infinite space. Thus, according to equation (5), *K* can be derived as follows:

7 $$\begin{array}{c}K=\frac{{N}_{{\rm{o}}}}{{\int }_{{T}_{{\rm{o}}}}^{{T}_{start}}(\sum _{{t}_{j} < t}\frac{{K}_{t}{e}^{\beta ({M}_{j}-{M}_{l})}}{{(t-{t}_{j}+c)}^{p}}){\rm{d}}t}=\frac{{N}_{{\rm{o}}}/{K}_{t}}{\sum _{i=2}^{{N}_{{\rm{o}}}}\sum _{{t}_{j} < {t}_{i}}{e}^{\beta ({M}_{j}-{M}_{l})}{{\rm I}}_{0}({t}_{i},{t}_{i-1},{t}_{j})+\sum _{{t}_{j} < {T}_{start}}{e}^{\beta ({M}_{j}-{M}_{l})}{{\rm I}}_{0}({T}_{start},{t}_{{N}_{{\rm{o}}}},{t}_{j})}\\ {{\rm I}}_{0}({t}_{i},{t}_{i-1},{t}_{j})={\int }_{{t}_{i-1}}^{{t}_{i}}\frac{{\rm{d}}t}{{(t-{t}_{j}+c)}^{p}}=\{\begin{array}{cc}[{({t}_{i}-{t}_{j}+c)}^{1-p}-{({t}_{i-1}-{t}_{j}+c)}^{1-p}]/(1-p) & p\ne 1\\ \mathrm{ln}[({t}_{i}-{t}_{j}+c)/({t}_{i-1}-{t}_{j}+c)] & p=1\end{array}\end{array}$$

Since the integral with respect to time in equation (7) cannot be calculated analytically over the interval [*T*_o_, *T*_*start*_], we approximated it by summing over the sub-intervals [*t*_*i*__−1_, *t*_*i*_] (where *i* = 2:*N*_o_) and the last interval [*t*_*N*__o_, *T*_*start*_] (where *t*_*N*__o_ is the arrival time of the *N*_o_^th^ event).

It is to note that parameters *K*, *K*_*t*_, and *K*_*R*_ are derived as a function of other model parameters in **θ**; therefore, the main parameters of the ETAS model include [*β*, *c*, *p*, *d*, *q*]. The rate of events in the ETAS model with magnitude (exactly) equal to *m*, denoted herein as *μ*_ETAS_ herein, is calculated by taking the derivative of equation (3) with respect to magnitude *m*:

8 $${\mu }_{{\rm{ETAS}}}(t,x,y,m|{\boldsymbol{\theta }},{\bf{s}}{\bf{e}}{{\bf{q}}}_{t},{M}_{l})=|\partial {\lambda }_{{\rm{ETAS}}}/\partial m|=\beta {e}^{-\beta (m-{M}_{l})}{\lambda }_{{\rm{ETAS}}}(t,x,y,{M}_{l}|{\boldsymbol{\theta }},{\bf{s}}{\bf{e}}{{\bf{q}}}_{t},{M}_{l})$$

### Robust estimation for the number of aftershock events

As mentioned above, **seq** denotes the sequence of events taking place before the beginning of the forecasting interval (i.e., in the interval [*T*_o_, *T*_*start*_)). However, the triggering effect of the events taking place during the forecasting interval [*T*_*start*_, *T*_*end*_] is expected to play a major role. The sequence of events taking place during the forecasting interval denoted as **seqg**, which is unknown at the time of forecasts, is simulated/generated herein. Let us assume that a plausible **seqg** is defined as the events within the forecasting interval defined as **seqg** = {(*IAT*_*i*_, *x*_*i*_, *y*_*i*_, *m*_*i*_), *T*_*start*_ ≤ *t*_*i*_ ≤ *T*_*end*_, *m*_*i*_ ≥ *M*_*l*_}, where *IAT*_i_ = *t*_*i*_ − *t*_*i*−1_ stands for the inter-arrival time. The robust estimate for the number of aftershock events in equation (2) should also consider all the plausible sequences of events **seqg** (i.e., the domain Ω_**seqg**_) that can happen during the forecasting time interval:

9 $$\begin{array}{rcl}N(x,y,m|{\bf{s}}{\bf{e}}{\bf{q}},{M}_{l}) & = & {N}_{b}(x,y,m|{M}_{l})\\  &  & +{\int }_{{{\rm{\Omega }}}_{{\boldsymbol{\theta }}}}[{\int }_{{{\rm{\Omega }}}_{{\bf{s}}{\bf{e}}{\bf{q}}{\bf{g}}}}({\int }_{{T}_{start}}^{{T}_{end}}\lambda (t,x,y,m|{\bf{s}}{\bf{e}}{\bf{q}}{\bf{g}},{\boldsymbol{\theta }},{\bf{s}}{\bf{e}}{\bf{q}},{M}_{l}){\rm{d}}t)\\  &  & \times p({\bf{s}}{\bf{e}}{\bf{q}}{\bf{g}}|{\boldsymbol{\theta }},{\bf{s}}{\bf{e}}{\bf{q}},{M}_{l}){\rm{d}}{\bf{s}}{\bf{e}}{\bf{q}}{\bf{g}}]p({\boldsymbol{\theta }}|{\bf{s}}{\bf{e}}{\bf{q}},{M}_{l}){\rm{d}}{\boldsymbol{\theta }}\end{array}$$

where *p*(**seqg**|**θ**, **seq**, *M*_*l*_) is the PDF for the generated sequence **seqg** given that **θ** and **seq** are known and *λ*(*t*, *x*, *y*, *m*|**seqg**, **θ**, **seq**, *M*_*l*_) is the space-time clustering ETAS model considering also the sequence of events taking place within the forecasting interval. The integral with respect to time in equation (9) cannot be calculated analytically over the entire interval [*T*_*start*_, *T*_*end*_], and is approximated by summing over the sub-intervals [*t*_*i*−1_, *t*_*i*_] within **seqg**:

10 $$\begin{array}{rcl}{\int }_{{T}_{start}}^{{T}_{end}}\lambda (t,x,y,m|{\bf{s}}{\bf{e}}{\bf{q}}{\bf{g}},{\boldsymbol{\theta }},{\bf{s}}{\bf{e}}{\bf{q}},{M}_{l}){\rm{d}}t & = & \sum _{{{\rm{\Omega }}}_{{\bf{s}}{\bf{e}}{\bf{q}}{\bf{g}}}}{\int }_{{t}_{i-1}}^{{t}_{i}}{\lambda }_{{\rm{ETAS}}}(t,x,y,m|{\bf{s}}{\bf{e}}{\bf{q}}{{\bf{g}}}_{i-1},{\boldsymbol{\theta }},{\bf{s}}{\bf{e}}{\bf{q}},{M}_{l}){\rm{d}}t\\  & = & \sum _{{{\rm{\Omega }}}_{{\bf{s}}{\bf{e}}{\bf{q}}{\bf{g}}}}(K{K}_{t}{K}_{R}{e}^{-\beta (m-{M}_{l})}\sum _{{t}_{j} < {t}_{i}}\frac{{e}^{\beta ({M}_{j}-{M}_{l})}{{\rm I}}_{0}({t}_{i},{t}_{i-1},{t}_{j})}{{({r}_{j}^{2}+{d}^{2})}^{q}})\end{array}$$

where *λ*_ETAS_ has the functional form presented in equation (3), and **seqg**_*i*−1_ is the previous (*i* − 1) events within the generated sequence. In the following sections, it is described first how sequence of events **seqg** for the forecasting interval is sampled based on *p*(**seqg**|**θ**, **seq**, *M*_*l*_). Later on, the method for sampling **θ** from the distribution *p*(**θ**|**seq**, *M*_*l*_) is discussed.

#### Generating sequences according to *p*(**seqg**|**θ**, **seq**, *M*_*l*_)

The probability distribution *p*(**seqg**|**θ**, **seq**, *M*_*l*_) can be written as follows (based on the probability product rule, see e.g. Jaynes^38^):

11 $$p({\bf{s}}{\bf{e}}{\bf{q}}{\bf{g}}|{\boldsymbol{\theta }},{\bf{s}}{\bf{e}}{\bf{q}},{M}_{l})=\prod _{i}p(IA{T}_{i},{x}_{i},{y}_{i},{M}_{i}|{\bf{s}}{\bf{e}}{\bf{q}}{{\bf{g}}}_{i-1},{\boldsymbol{\theta }},{\bf{s}}{\bf{e}}{\bf{q}},{M}_{l})$$

where **seqg**_*i*_ is the generated sequence up to the *i*th event, where **seqg**_*i*_ = {**seqg**_*i*−1_, (*IAT*_*i*_, *x*_*i*_, *y*_*i*_, *m*_*i*_)}, and the sequence of events that precede the *i*th generated event is {**seq**, **seqg**_*i*−1_}. The probability distribution *p*(*IAT*_*i*_, *x*_*i*_, *y*_*i*_, *m*_*i*_|**seqg**_*i*−1_, **θ**, **seq**, *M*_*l*_) can be further expanded (again using the probability product rule) as follows:

12 $$\begin{array}{rcl}p(IA{T}_{i},{x}_{i},{y}_{i},{m}_{i}|{\bf{s}}{\bf{e}}{\bf{q}}{{\bf{g}}}_{i-1},{\boldsymbol{\theta }},{\bf{s}}{\bf{e}}{\bf{q}},{M}_{l}) & = & p({m}_{i}|{\bf{s}}{\bf{e}}{\bf{q}}{{\bf{g}}}_{i-1},{\boldsymbol{\theta }},{\bf{s}}{\bf{e}}{\bf{q}},{M}_{l})\\  & = & p(IA{T}_{i}|{m}_{i},{\bf{s}}{\bf{e}}{\bf{q}}{{\bf{g}}}_{i-1},{\boldsymbol{\theta }},{\bf{s}}{\bf{e}}{\bf{q}},{M}_{l})\\  & = & p({x}_{i},{y}_{i}|IA{T}_{i},{m}_{i},{\bf{s}}{\bf{e}}{\bf{q}}{{\bf{g}}}_{i-1},{\boldsymbol{\theta }},{\bf{s}}{\bf{e}}{\bf{q}},{M}_{l})\end{array}$$

where *p*(*m*_*i*_|**seqg**_*i*−1_, **θ**, **seq**, *M*_*l*_) is the marginal PDF for the magnitude *m*_*i*_ given the sequence of events that precede it, **θ**, and *M*_*l*_; *p*(*IAT*_*i*_|*m*_*i*_, **seqg**_*i*−1_, **θ**, **seq**, *M*_*l*_) is the (conditional) marginal PDF for inter-arrival time *IAT*_*i*_ given that the value of magnitude is equal to *m*_*i*_; finally, the term *p*(*x*_*i*_, *y*_*i*_|*IAT*_*i*_, *m*_*i*_, **seqg**_*i*−1_, **θ**, **seq**, *M*_*l*_) is the conditional joint PDF for the spatial position (*x*_*i*_, *y*_*i*_) ∈ *A* given that *IAT*_*i*_ and *m*_i_ are known. It should be noted that the break-down into the product of several conditional PDFs is necessary during the sequence generation process.

To generate a plausible sequence of events during the forecasting interval, the procedure, illustrated by the flowchart in Supplementary Fig. S4, is adopted. The *i*th event within the sequence **seqg** is generated through the following steps:

1. Generate the magnitude of the *i*th event, *m*_*i*_, within **seqg** according to the following truncated Exponential PDF with rate *β* ^23^ (see Phase 1 in Supplementary Fig. S4).
  13 $$p(m|{\bf{s}}{\bf{e}}{\bf{q}}{{\bf{g}}}_{i-1},{\boldsymbol{\theta }},{\bf{s}}{\bf{e}}{\bf{q}},{M}_{l})\cong p(m|{\boldsymbol{\theta }},{\bf{s}}{\bf{e}}{\bf{q}},{M}_{l})=\frac{\beta {e}^{-\beta m}}{{e}^{-\beta {M}_{l}}-{e}^{-\beta {M}_{\max }}}$$
  where *M*_max_ is the upper-bound magnitude of the site. This truncated Exponential PDF has a cumulative density function equal to $\frac{1-\exp (-\beta (m-{M}_{l}))}{{F}_{{M}_{{\rm{\max }}}}}\triangleq \frac{1-\exp (-\beta (m-{M}_{l}))}{1-\exp (-\beta ({M}_{{\rm{\max }}}-{M}_{l}))}$; hence, a generated/sampled *m*_*i*_ can analytically be drawn as ${m}_{i}=\frac{-\mathrm{log}(1-{F}_{{M}_{{\rm{\max }}}}\cdot {r}_{{\rm{rand}}})}{\beta }+{M}_{l}$ where *r*_rand_ (as shown also in the flowchart of Supplementary Fig. S4) is a random number generated from a Uniform distribution in the interval (0, 1).
2. Generate the inter-arrival time of the ith event within seqg (given that its magnitude mi is already known from previous step) by using the Thinning algorithm^39, 40^. The algorithm, as shown in Phase 2 in Supplementary Fig. S4, consists of a two-stage process. First, the temporal Poisson rate over the entire aftershock zone is calculated for an event with magnitude equal to *m*_*i*_ at time *t* = *t*_*i*_ − 1, and denoted by *μ*_max_ using equation (8) as follows:
  14 $${\mu }_{\max }={\iint }_{x,y\in A}{\mu }_{{\rm{ETAS}}}({t}_{i-1},x,y,{m}_{i}|{\bf{s}}{\bf{e}}{\bf{q}}{{\bf{g}}}_{i-1},{\boldsymbol{\theta }},{\bf{s}}{\bf{e}}{\bf{q}},{M}_{l}){\rm{d}}x{\rm{d}}y$$
  Note that for generating the first event within the forecasting interval [*T*_*start*_, *T*_*end*_], *t*_*i*−1_ is set to *T*_*start*_. In the next stage of Thinning algorithm, the inter-arrival time *IAT*_gen_ is sampled/generated from a homogeneous Exponential PDF with the form ${\mu }_{{\rm{\max }}}\exp (-{\mu }_{{\rm{\max }}}IAT)$ which is equivalent to generating it as $IA{T}_{{\rm{gen}}}=\frac{-\,\mathrm{log}(1-{r}_{{\rm{rand}}})}{{\mu }_{{\rm{\max }}}}$. The Poisson rate at time *t*_gen_ = *t*_*i*−1_ + *IAT*_gen_ and denoted by *μ*_gen_ can then be calculated from equation (14) by substituting *t*_*i*−1_ with *t*_gen_. The generated inter-arrival time can then be (1) either accepted with probability *p* $\,=\frac{{\mu }_{{\rm{gen}}}}{{\mu }_{{\rm{\max }}}}$ and thus *t*_*i*_ = *t*_gen_ and the procedures continues by generating the next interarrival time until *t*_i_ > *T*_*end*_; (2) or rejected with probability 1-*p*. Let us denote the rejected *t*_gen_ as *t*_gen_^(−)^ (in order to keep track of it for the next simulation). Also in the case of rejection, the procedure continues by sampling a new inter-arrival time *IAT*_gen_ from the homogeneous Exponential PDF with rate *μ*_max_. The new generated arrival time is calculated as *t*_gen_ = *t*_gen_^(−)^ + *IAT*_gen_, *t*_gen_ < *T*_*end*_. The quantities *μ*_gen_ and *p* are calculated again to test whether the newly generated interarrival time is accepted or rejected.
3. Generate/sample the Cartesian coordinates (*x*_*i*_, *y*_*i*_) for the *i*th event, given that the magnitude *m*_*i*_, the time of occurrence *t*_*i*_, and the previous (*i* − 1) events within the generated sequence **seqg**_*i*-1_ are known (see Supplementary Fig. S4, Phase 3), according the following joint PDF using the probability product rule:

15 $$p(x,y|IA{T}_{i},{m}_{i},{\bf{s}}{\bf{e}}{\bf{q}}{{\bf{g}}}_{i-1},{\boldsymbol{\theta }},{\bf{s}}{\bf{e}}{\bf{q}},{M}_{l})=\frac{p(IA{T}_{i},x,y,{m}_{i}|{\bf{s}}{\bf{e}}{\bf{q}}{{\bf{g}}}_{i-1},{\boldsymbol{\theta }},{\bf{s}}{\bf{e}}{\bf{q}},{M}_{l})}{{\iint }_{x,y\in A}p(IA{T}_{i},x,y,{m}_{i}|{\bf{s}}{\bf{e}}{\bf{q}}{{\bf{g}}}_{i-1},{\boldsymbol{\theta }},{\bf{s}}{\bf{e}}{\bf{q}},{M}_{l}){\rm{d}}x{\rm{d}}y}$$

The nominator *p*(*IAT*_*i*_, *x*, *y*, *m*_*i*_|**seqg**_*i*−1_, **θ**, **seq**, *M*_*l*_) in equation (15) can be calculated as:

16 $$\begin{array}{ccc}p(IA{T}_{i},x,y,{m}_{i}|{\bf{s}}{\bf{e}}{\bf{q}}{{\bf{g}}}_{i-1},{\boldsymbol{\theta }},{\bf{s}}{\bf{e}}{\bf{q}},{M}_{l}) & = & {\mu }_{{\rm{E}}{\rm{T}}{\rm{A}}{\rm{S}}}({t}_{i},x,y,{m}_{i}|{\bf{s}}{\bf{e}}{\bf{q}}{{\bf{g}}}_{i-1},{\boldsymbol{\theta }},{\bf{s}}{\bf{e}}{\bf{q}},{M}_{l})\\  &  & {\times e}^{-{\int }_{{t}_{i-1}}^{{t}_{i}}{\mu }_{{\rm{E}}{\rm{T}}{\rm{A}}{\rm{S}}}(t,x,y,{m}_{i}|{\bf{s}}{\bf{e}}{\bf{q}}{{\bf{g}}}_{i-1},{\boldsymbol{\theta }},{\bf{s}}{\bf{e}}{\bf{q}},{M}_{l}){\rm{d}}t}\end{array}$$

where *μ*_ETAS_ is calculated from equation (8), and the integral in the sub-interval [*t*_*i*−1_, *t*_*i*_] has the similar analytical expression shown in equation (10) being multiplied by *β* in order to account for *μ*_ETAS_.

#### Sampling **θ** from the distribution *p*(**θ**|**seq**, *M*_*l*_)

The probability distribution *p*(**θ**|**seq**, *M*_*l*_) can be calculated using Bayesian parameter estimation:

17 $$p({\boldsymbol{\theta }}|{\bf{s}}{\bf{e}}{\bf{q}},{M}_{l})={C}^{-1}p({\bf{s}}{\bf{e}}{\bf{q}}|{\boldsymbol{\theta }},{M}_{l})p({\boldsymbol{\theta }}|{M}_{l})$$

where *p*(**seq**|**θ**, *M*_*l*_) denotes the likelihood of the observed sequence given the vector of model parameters **θ** and lower cut-off magnitude *M*_*l*_, *p*(**θ**|*M*_*l*_) is the prior distribution for the vector **θ**, and *C* ^−1^ is a normalizing constant. In lieu of additional information (e.g., statistics of regional model parameters), the prior joint distribution *p*(**θ**|*M*_*l*_) can be estimated as the product of marginal uniform probability distributions for each model parameter. In order to sample from *p*(**θ**|**seq**, *M*_*l*_), Markov Chain Monte Carlo (MCMC) simulation routine is employed which is particularly useful for cases where the sampling needs to be done from a probability distribution that is known up to a constant value^27^ (herein, *C* ^−1^). The MCMC routine uses the Metropolis-Hastings (MH) algorithm^41, 42^ in order to generate samples as a Markov Chain sequence used first to sample from the target probability distribution *p*(**θ**|**seq**, *M*_*l*_), and later to estimate the robust reliability in equation (9). The MH routine, as shown in Supplementary Fig. S5, functions by generating a Markov chain that produces a sequence of samples [**θ**_1_→**θ**_2_→…→**θ**_*n*_→…], where **θ**_*n*_ represents the state of Markov chain at *n*th iteration (the first few samples are often discarded to reduce the initial transient effect). This procedure continues until the *ns* Markov chain samples were simulated. It can be shown that the samples from the chain after the initial transient ones reflect samples from the target distribution *p*(**θ**|**seq**, *M*_*l*_). Given the Markovian nature of this simulation scheme, we limit ourselves to describe how the (*n* + 1)th sample **θ**_*n*+1_ is generated given that the *n*th sample **θ**_*n*_ is already known:

- Generate a candidate sample **θ** ^*^ from a proposal (candidate) distribution *ζ*(**θ**|**θ**_*n*_). It is important to note that there are no specific restrictions about the choice of *ζ* (·) apart from the fact that it should be possible to calculate both *ζ* (**θ**_*i*__+__1_|**θ**_*i*_) and *ζ* (**θ**_*i*_|**θ**_*i*__+__1_).
- Accept the candidate sample with the probability *p*_accept_ = min(1, *r*) (where *r* is defined in equation (18) as follows) and set **θ**_*n*+1_ = **θ** ^*^; otherwise, **θ**_*n*+1_ = **θ**_*n*_:

18 $$r=\frac{p({{\boldsymbol{\theta }}}^{\ast }|{\bf{s}}{\bf{e}}{\bf{q}},{M}_{l})}{p({{\boldsymbol{\theta }}}_{n}|{\bf{s}}{\bf{e}}{\bf{q}},{M}_{l})}\cdot \frac{\zeta ({{\boldsymbol{\theta }}}_{n}|{{\boldsymbol{\theta }}}^{\ast })}{\zeta ({{\boldsymbol{\theta }}}^{\ast }|{{\boldsymbol{\theta }}}_{n})}=(\frac{p({\bf{s}}{\bf{e}}{\bf{q}}|{{\boldsymbol{\theta }}}^{\ast },{M}_{l})p({{\boldsymbol{\theta }}}^{\ast }|{M}_{l})}{p({\bf{s}}{\bf{e}}{\bf{q}}|{{\boldsymbol{\theta }}}_{n},{M}_{l})p({{\boldsymbol{\theta }}}_{n}|{M}_{l})})\cdot \frac{\zeta ({{\boldsymbol{\theta }}}_{n}|{{\boldsymbol{\theta }}}^{\ast })}{\zeta ({{\boldsymbol{\theta }}}^{\ast }|{{\boldsymbol{\theta }}}_{n})}$$

It can be shown^27^ using the Total probability theorem that, if the current sample **θ**_*n*_ is distributed as *p*(·|**seq**, *M*_*l*_), also the (*n* + 1)th sample **θ**_*n*+1_ is distributed as *p*(·|**seq**, *M*_*l*_):

19 $$\begin{array}{rcl}PDF({{\boldsymbol{\theta }}}_{n+1}|{\bf{s}}{\bf{e}}{\bf{q}},{M}_{l}) & = & {\int }_{{\Omega }_{{{\boldsymbol{\theta }}}_{n}}}p({{\boldsymbol{\theta }}}_{n+1}|{{\boldsymbol{\theta }}}_{n})\mathop{\underbrace{p({{\boldsymbol{\theta }}}_{n}|{\bf{s}}{\bf{e}}{\bf{q}},{M}_{l})}}\limits_{p({{\boldsymbol{\theta }}}_{n})}{\rm{d}}{{\boldsymbol{\theta }}}_{n}\\  & = & {\int }_{{\Omega }_{{{\boldsymbol{\theta }}}_{n}}}\min (1,r)\zeta ({{\boldsymbol{\theta }}}_{n+1}|{{\boldsymbol{\theta }}}_{n})p({{\boldsymbol{\theta }}}_{n}|{\bf{s}}{\bf{e}}{\bf{q}},{M}_{l}){\rm{d}}{{\boldsymbol{\theta }}}_{n}\\  & = & {\int }_{{\Omega }_{{{\boldsymbol{\theta }}}_{n}}}\min (1,{r}^{-1})\zeta ({{\boldsymbol{\theta }}}_{n}|{{\boldsymbol{\theta }}}_{n+1})p({{\boldsymbol{\theta }}}_{n+1}|{\bf{s}}{\bf{e}}{\bf{q}},{M}_{l}){\rm{d}}{{\boldsymbol{\theta }}}_{n}\\  & = & p({{\boldsymbol{\theta }}}_{n+1}|{\bf{s}}{\bf{e}}{\bf{q}},{M}_{l}){\int }_{{\Omega }_{{{\boldsymbol{\theta }}}_{n}}}p({{\boldsymbol{\theta }}}_{n}|{{\boldsymbol{\theta }}}_{n+1}){\rm{d}}{{\boldsymbol{\theta }}}_{n}=p({{\boldsymbol{\theta }}}_{n+1}|{\bf{s}}{\bf{e}}{\bf{q}},{M}_{l})\end{array}$$

Equation (19) is derived using the arithmetic property that for any positive numbers *A* and *B*, the identity min(1, *A*/*B*)∙*B* = min(1, *B*/*A*)∙*A* holds. Having the proposal PDF *ζ* centered around the current sample renders the MH algorithm similar to a local random walk that adaptively leads to the generation of the target PDF. In order to improve the rate of convergence of the simulation process, we have used an adaptive MH algorithm (as proposed by Beck and Au^27^) that introduces a sequence of intermediate evolutionary candidate PDF’s that resemble more and more the target PDF.

#### Calculating the likelihood of the observed sequence *p*(**seq**|**θ**, *M*_*l*_)

The likelihood for the observed sequence, **seq** = {(*t*_*i*_, *x*_*i*_, *y*_*i*_, *m*_*i*_), *t*_*i*_ < *T*_*start*_, *m*_*i*_ ≥ *M*_*l*_, *i* = 1: *N*_o_}, with *N*_o_ events, including the mainshock (with *i* = 1) and the sequence of aftershocks, can be calculated as:

20 $$\begin{array}{rcl}p({\bf{s}}{\bf{e}}{\bf{q}}|{\boldsymbol{\theta }},{M}_{l}) & = & (\prod _{i=2}^{{N}_{{\rm{o}}}}p(IA{T}_{i},{x}_{i},{y}_{i},{m}_{i}|{\boldsymbol{\theta }},{\bf{s}}{\bf{e}}{{\bf{q}}}_{i-1},{M}_{l}))P(IA{T}_{{N}_{{\rm{o}}}+1} > {T}_{start}-{t}_{{N}_{{\rm{o}}}}|{\boldsymbol{\theta }},{\bf{s}}{\bf{e}}{\bf{q}},{M}_{l})\\  & = & \prod _{i=2}^{{N}_{{\rm{o}}}}({\mu }_{{\rm{ETAS}}}({t}_{i},{x}_{i},{y}_{i},{m}_{i}|{\boldsymbol{\theta }},{\bf{s}}{\bf{e}}{{\bf{q}}}_{i-1},{M}_{l}){e}^{-{\int }_{{t}_{i-1}}^{{t}_{i}}{\mu }_{{\rm{ETAS}}}(t,{x}_{i},{y}_{i},{m}_{i}|{\boldsymbol{\theta }},{\bf{s}}{\bf{e}}{{\bf{q}}}_{i-1},{M}_{l}){\rm{d}}t}){\times e}^{-{\int }_{{t}_{{N}_{{\rm{o}}}}}^{{T}_{start}}{\iint }_{x,y\in A}{\lambda }_{{\rm{ETAS}}}(t,x,y,{M}_{l}|{\boldsymbol{\theta }},{\bf{s}}{\bf{e}}{\bf{q}}){\rm{d}}x{\rm{d}}y{\rm{d}}t}\end{array}$$

where λ(∙) and *μ*(∙) are ETAS rates calculated from equations (3) and (8), respectively. The first term of the likelihood function in equation (20) is calculated by multiplying the probabilities that the *i*th arrival time (*i* = 2:*N*_o_) is equal to the observed value, *t*_*i*_ = *t*_*i*−1_ + *IAT*_*i*_, occurred at spatial position (*x*_*i*_, *y*_*i*_) ∈ *A* with magnitude *m*_*i*_ assuming a non-homogenous Poisson process (i.e., the inter-arrival times are independent) with a rate equal to *μ*_ETAS_ (equation 8). Accordingly

20 $${\int }_{{t}_{i-1}}^{{t}_{i}}{\mu }_{{\rm{ETAS}}}(t,{x}_{i},{y}_{i},{m}_{i}|{\boldsymbol{\theta }},{\bf{s}}{\bf{e}}{{\bf{q}}}_{i-1},{M}_{l}){\rm{d}}t=K{K}_{t}{K}_{R}\beta {e}^{-\beta (m-{M}_{l})}\sum _{{t}_{j} < {t}_{i}}\frac{{e}^{\beta ({M}_{j}-{M}_{l})}{{\rm I}}_{0}({t}_{i},{t}_{i-1},{t}_{j})}{{({r}_{ji}^{2}+{d}^{2})}^{q}}$$

where the term I_0_ is previously defined in equation (7) and *r*_*ji*_ indicates the distance of the *i*th event to the previously occurred *j*th event. The last probability term in equation (20) is the probability that no event with magnitude greater than the cut-off level *M*_*l*_ takes place over the entire aftershock zone ***A*** in the time interval between the *N*_o_^th^ event at *t*_*N*o_ and *T*_*start*_.

## Electronic supplementary material

Supplementary Information
