# Supplementary material for: Robust seismicity forecasting based on Bayesian parameter estimation for epidemiological spatio-temporal aftershock clustering models
Source: Sci Rep. 2017 Aug 29;7:9803. doi: 10.1038/s41598-017-09962-z (PMC5575305; doi:10.1038/s41598-017-09962-z)
Supplement: Supplementary file 1 — Supplementary Information [file 41598_2017_9962_MOESM1_ESM.pdf]

Supplementary Information

Robust seismicity forecasting based on Bayesian parameter estimation for epidemiological spatio-temporal aftershock clustering models

Hossein Ebrahimian[1] **& Fatemeh Jalayer**[2,*]

[1] Post-doctoral Researcher, Department of Structures for Engineering and Architecture, University of Naples Federico II, Via Claudio 21, 80125 Naples, Italy

[2] Associate Professor, Department of Structures for Engineering and Architecture, University of Naples Federico II, Via Claudio 21, 80125 Naples, Italy, Correspondence and requests for materials should be addressed to F.J. (email: fatemeh.jalayer@unina.it)

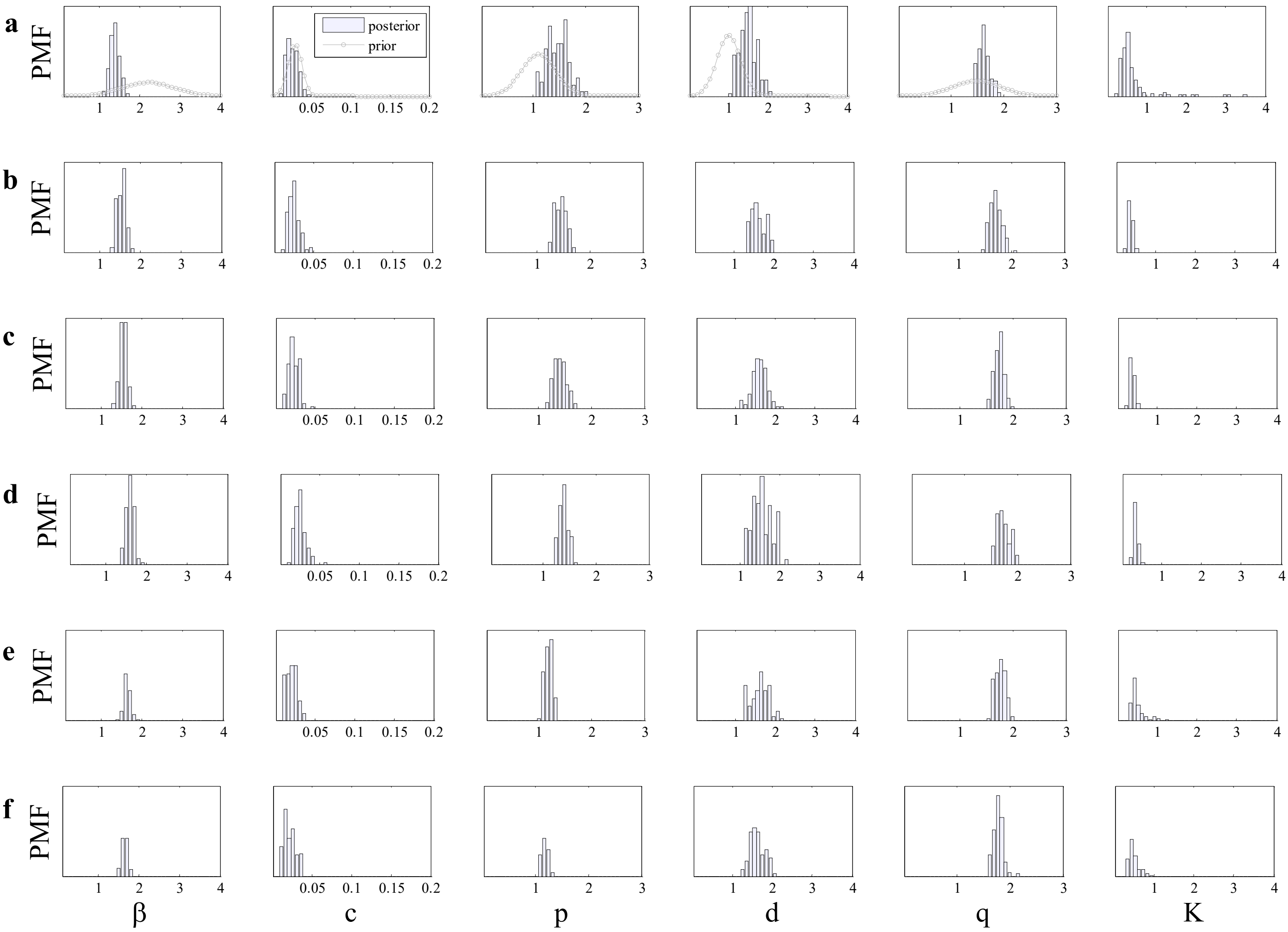

Figure S1. The sampled histograms representing marginal prior and posterior PDF's for the six model parameters θ=[β, K, c, p, d, q] based on MCMC simulation, (a) August 24, (b) August 25, (c) August 26, (d) August 28, (e) September 02, (f) September 03. The corresponding statistics (mean and COV) are reported in Table 1 of the manuscript. (MATLAB 2016b, http://softwaresso.unina.it/matlab/ is used to create this figure.)

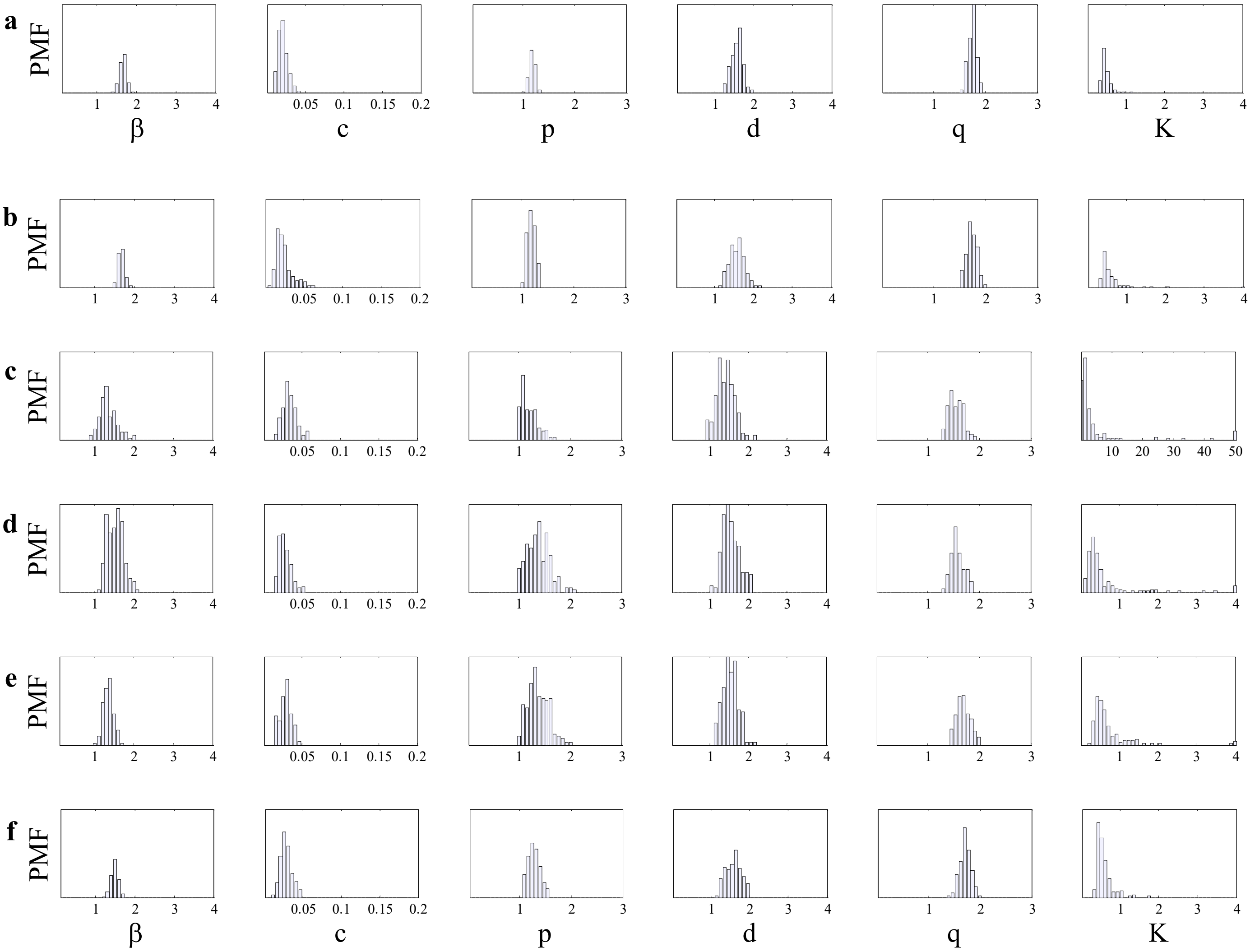

Figure S2. The sampled histograms representing marginal prior and posterior PDF's for the six model parameters θ=[β, K, c, p, d, q] based on MCMC simulation, (a) prior, (b) October 26, T_{start}=06:00UTC and October 26, T_{start}=18:00UTC, (c) October 26, T_{start}=20:00UTC, (d) October 26, T_{start}=24:00UTC, (e) October 27, (f) October 29. The corresponding statistics (mean and COV) are reported in Table 2 of the manuscript. (MATLAB 2016b, http://softwaresso.unina.it/matlab/ is used to create this figure.)

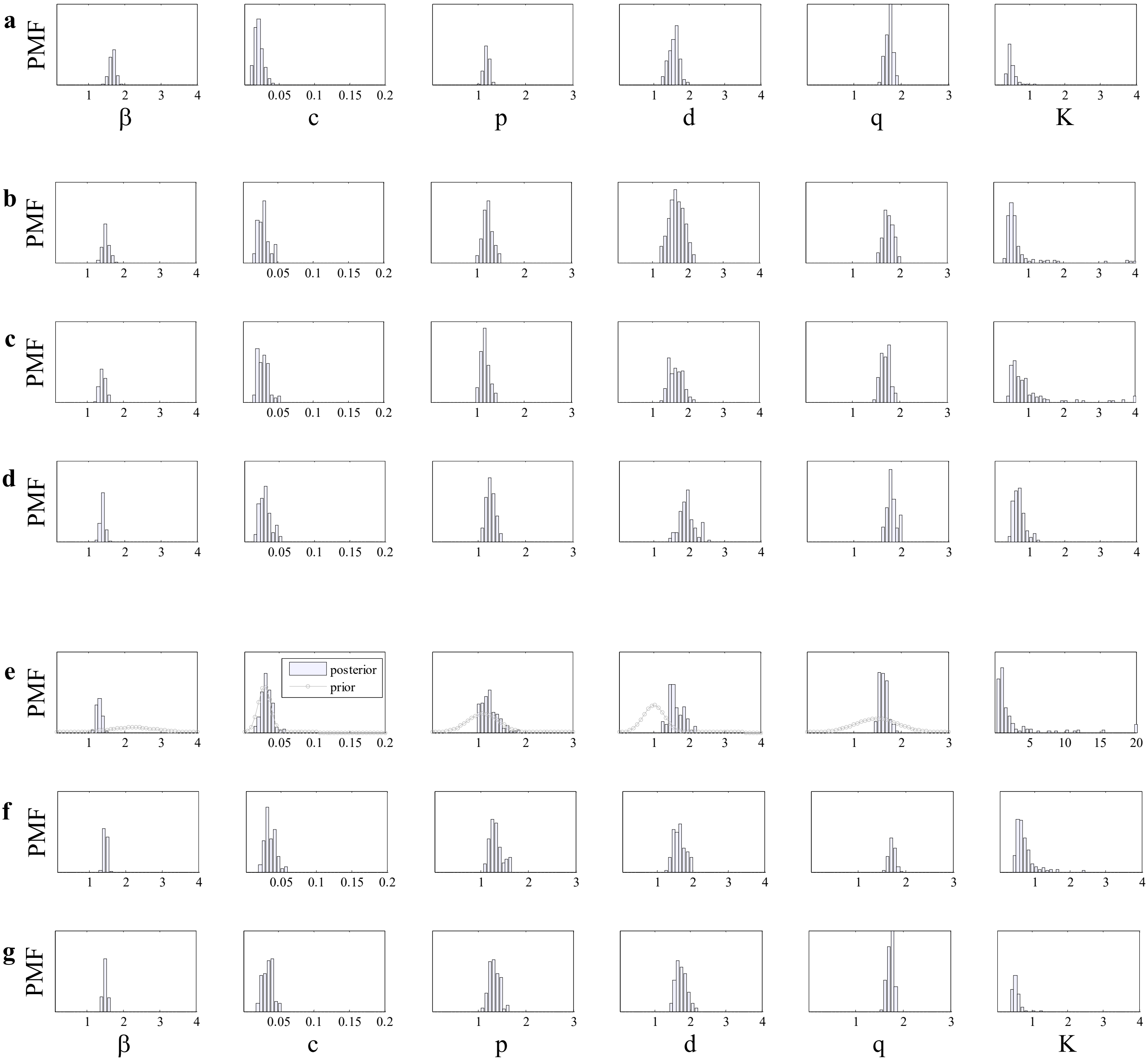

Figure S3. The sampled histograms representing marginal prior and posterior PDF's for the six model parameters θ=[β, K, c, p, d, q] based on MCMC simulation, (a) Prior for the next three posterior distributions, (b) October 30, T_{start}=06:00UTC, (c) October 30, T_{start}=7:00UTC, (d) October 30, T_{start}=12:00UTC (see Figure 5c), (e) October 30, T_{start}=12:00UTC (see Figure 5d) together with the priors (these priors are also used for the next two posteriors), (f) October 31, (g) November 1. The corresponding statistics (mean and COV) are reported in Table 3 of the manuscript. (MATLAB 2016b, http://softwaresso.unina.it/matlab/ is used to create this figure.)

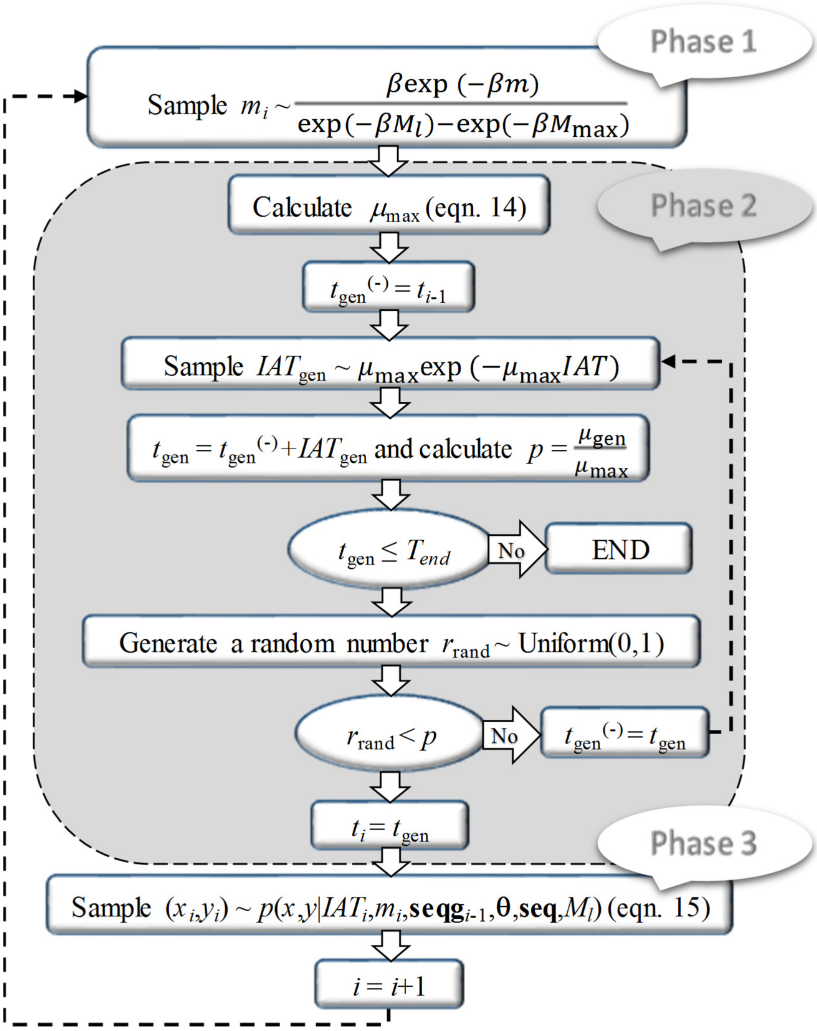

Figure S4. The flowchart for generating **seqg**

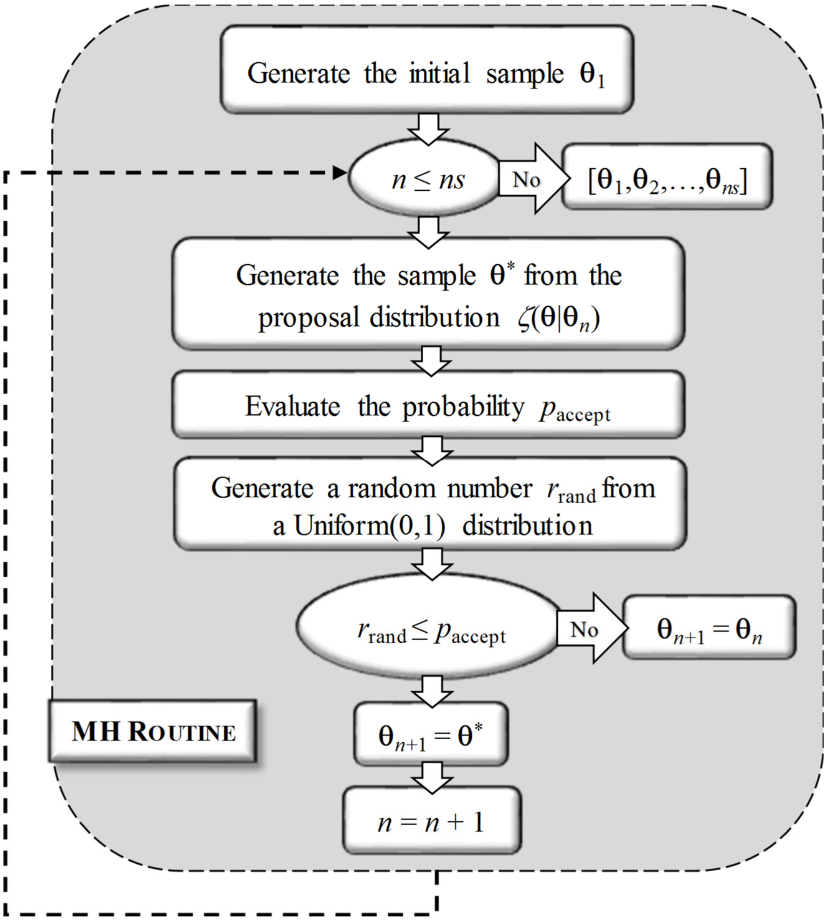

Figure S5. Metropolis-Hastings (MH) algorithm

Discussion on the Completeness Magnitude M_c

In order to address the issue of catalogue incompleteness explicitly, we checked the completeness magnitude, M_c, at the beginning of each forecasting interval so that the desired lower cut-off magnitude M_l be always greater than or equal to M_c, i.e., $M_l \geq M_c$. It is to note that M_l is an important input data for the automated forecasting procedure presented in the manuscript. The issue of magnitude incompleteness seems to be more critical when providing early forecasts in the immediate aftermath of a main seismic event. This can be attributed both to the lack of data in the short time elapsed after the main event and the missing data in certain magnitude ranges. Nevertheless, as more time passes and the observation history at the time of forecast starts to become more populated, the magnitude incompleteness seems to be less critical. Herein, we have employed two alternative methods to check how the completeness magnitude varies through time:

1. The direct use of frequency-magnitude distribution plot of the aftershock events available at the time of issuing the forecast (denoted as the observation history **seq** in the manuscript): the normal trend in frequency-magnitude curve has an approximately exponential decrease as the magnitude increases (i.e., linearly decreasing in logarithmic scale following a Gutenberg-Richter relationship as $\log_{10}[N(M \geq m)] = a_{reg} - b_{reg} \cdot m$ where N stands for the number of aftershock with magnitudes equal to or greater than m, and $[a_{reg}, b_{reg}]$ are regression coefficients). In case that the data is incomplete, a flattening in a certain lower magnitude range (having higher frequencies) can be monitored. Accordingly, the completeness magnitude denoted as M_c is visually picked up as the point where the magnitude-frequency curve becomes approximately linear in the semi-logarithmic scale.

2. Using a Bayesian updating approach for calculating the b-value versus various magnitude thresholds: the method detects the magnitude threshold where the mode (maximum likelihood) for the posterior probability distribution of b, denoted as b_{ML} herein, becomes roughly invariant. This magnitude threshold can be interpreted as M_c (see also Ebrahimian et al.[23]). The posterior probability distribution for b given the observed history up to the time of forecasting, denoted as **seq** in the manuscript, and the lower cut-off magnitude M_l, denoted as $p(b|\mathbf{seq},M_l)$ can be determined according to the Bayes's theorem as:

$$p\left(b\middle|\mathbf{seq},M_l\right)=c^{-1}p\left(\mathbf{seq}\middle|b,M_l\right)p\left(b\right)=c^{-1}\left(\prod_i \frac{\beta e^{-\beta m_i}}{e^{-\beta M_l}-e^{-\beta M_u}}\right)p\left(b\right) \qquad \text{(S1)}$$

where c^{-1} is the normalizing constant of the Bayes's expression; $p(\mathbf{seq}|b,M_l)$ is the likelihood function for magnitude data m_i in the **seq** given b and lower cut-off magnitude M_l; $p(b|M_l)\approx p(b)$ is the prior probability distribution. Herein, we use a Lognormal probability distribution to define the prior $p(b)$ having median equal to the b-value suggested by Lolli and Gasperini[33] (for the Italian generic model parameter) and COV equal to 0.30.

Figure S6 (a) (left column) illustrates the frequency-magnitude distribution plot and the procedure for selecting M_c based on method 1 explained above. The completeness magnitude M_c (drawn by red-dashed vertical line) is identified (visually) as the point beyond which the magnitude-frequency curve in terms of pairs $\{m, \log_{10}[N(M\geq m)]\}$ starts to show a linear trend (in the logarithmic scale). Figure S6(b) (right column) represents the procedure defined as method 2. It plots the maximum likelihood estimate (i.e., the mode) for the posterior distribution $p(b|\mathbf{seq},M_l)$, denoted as b_{ML}, with respect to various magnitude thresholds. The completeness magnitude M_c is identified as the magnitude threshold associated with the point after which the b_{ML}-values become roughly invariant (i.e., before reaching M_c, the b_{ML}-values are showing an increasing trend). Each subfigure corresponds to the forecasting interval stated in the figure title. It is also noteworthy that

the catalogue of registered events used in this study contains all the aftershocks above or equal to the threshold magnitude 2.0. In the following subfigures, where M_c is less than 2.0, no red-dashed vertical line is drawn. Moreover, the black-dotted vertical line represents the lower cut-off magnitude M_l for the desired forecasting interval.

The first forecasting time interval of 1-day starting at T_{start} = 6:00 UTC of 24 August 2016 (see also Figure 2a) is particularly critical in terms of assigning M_c since the **seq** consists of limited data (i.e., events taken place in the few hours elapsed after the main-shock up to T_{start}). The magnitude of completeness, $M_c \approx 2.70$ is less than the lower cut-off magnitude M_l=3.0 according to the plots in the first row of Figure S6 (Methods 1 and 2 described above). Note that the values b_{reg} and b_{ML} and also the regression line (i.e., the grey solid line) shown in Figure S6(a) are just reported for illustrative purposes. Both procedures are primarily helpful in visualizing and monitoring the appropriate point for selecting M_l.

The second row of Figure S6 corresponds to the second forecasting time interval of 1-day starting at T_{start} = 6:00 UTC of 25 August 2016 (see also Figure 2b). It is revealed that the completeness threshold of the catalog of aftershocks can be set to magnitudes lower than 2.0 (not shown in the figure, as noted previously). As a result (as also mentioned in the manuscript), for the first part of the catalog from August 24 up to October 25, we establish the magnitude threshold M_l=3.0 although M_c<3.0. This choice is motivated by two reasons: (1) events with magnitudes lower than 3.0 are not expected to significantly affect the built environment; (2) the computational effort for updating the parameters of the ETAS model, especially in the subsequent time intervals, can be significantly reduced by choosing a cut-off magnitude larger than the completeness threshold.

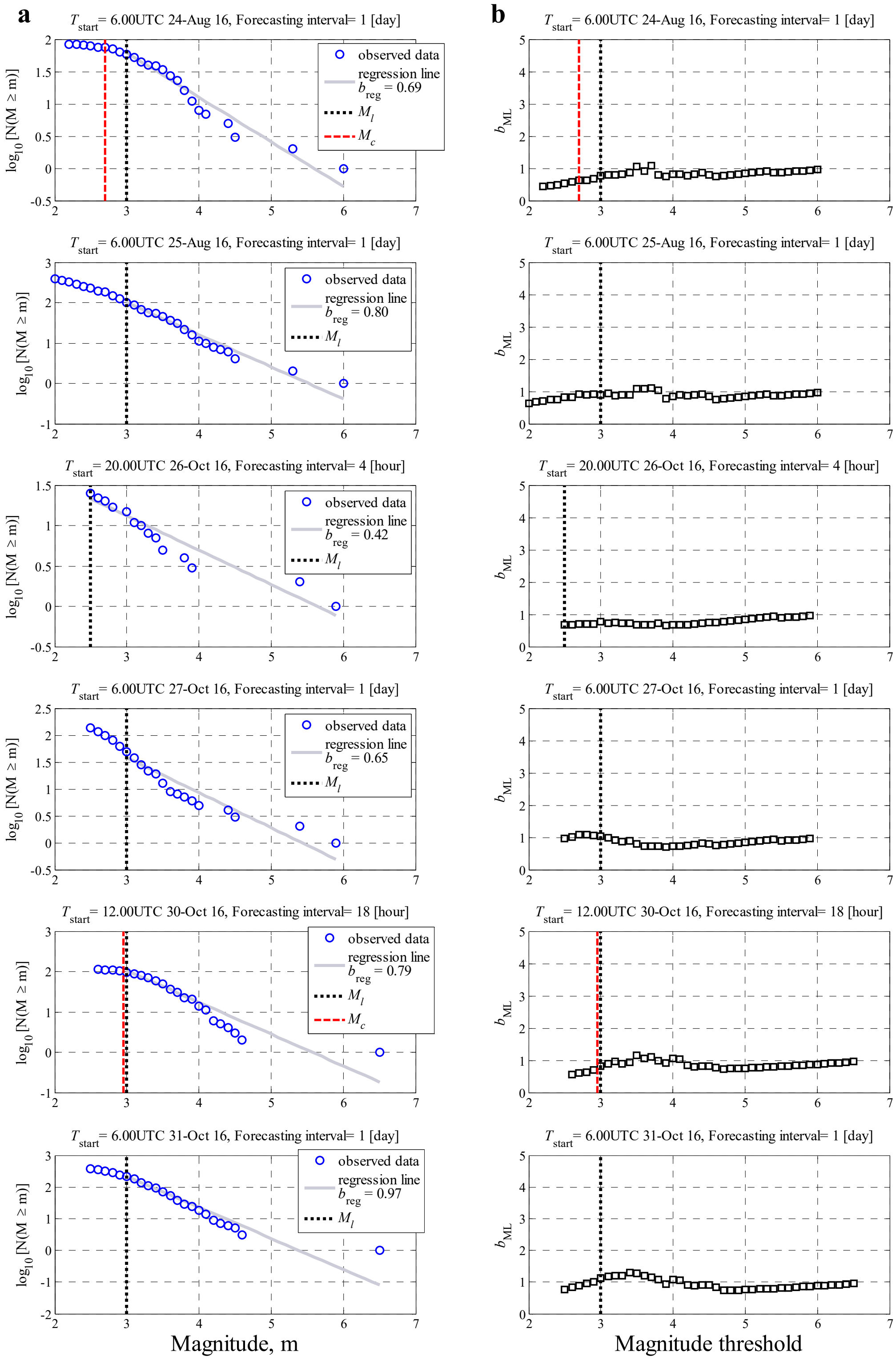

Figure S6. (a) The frequency-magnitude distribution of aftershocks from the time elapsed after the main-shock up to T_{start}; (b) Lower cut-off magnitude versus the estimated b_{ML}-value through Bayesian updating. The red dashed and the black dotted vertical lines in the sub-figures represent the completeness magnitude M_c and the lower cut-off threshold M_l, respectively (MATLAB 2016b, http://softwaresso.unina.it/matlab/ is used to create this figure.)

Focusing on the second sub-sequence from October 26 up to October 29, the 3rd row of Figure S6 represents the completeness calculation regarding the 4-hour forecasting from 20:00 UTC of October 26 (see also Figure 3c). With reference to the manuscript, we shift the time of origin by setting T_o to 17:10 UTC of 26th of October. Therefore, the sequence of events **seq** includes all the triggered events occurred after 17:10 UTC of 26/10/2016 (including the main Mw 5.4 event). For this specific forecast, the lower cut-off magnitude is set to M_l=2.5 in order to gain more data for model updating purposes. In this case, no specific value for M_c can be detected. The 4th row of Figure S6 shows the proper choice of the lower cut-off magnitude M_l=3.0 (again, in the absence of any indication for M_c) for the 24-hour forecasting from 6:00 UTC of 27th of October (see Figure 3e).

For the third sub-sequence starting from October 30 up to November 1, the completeness magnitude is verified for the forecasting of 30/10/2016 with T_{start} set to 12:00UTC and T_{end} set to 06:00 UTC of 31/10/2016 (i.e., 18-hour interval, see Figure 5d). As mentioned in the manuscript, we performed a shift in the time of origin T_o from 17:10 UTC of 26th October to 6:40 UTC of 30th October (the time of the main Mw 6.5 event). The 5th row of Figure S6 establishes M_c=M_l=3.0 (i.e., the lower cut-off magnitude is set equal to the completeness magnitude). Finally, the 6th row in Figure S6 shows the completeness check for the 24-hour forecasting from 6:00 UTC of October 31st (see Figure 5e) indicating the adequacy of M_l=3.0 in the absence of any indications for M_c.
